# The muscle-relaxing C-terminal peptide from troponin I populates a nascent helix, facilitating binding to tropomyosin with a potent therapeutic effect

**DOI:** 10.1074/jbc.RA120.016012

**Published:** 2021-01-07

**Authors:** Felipe Hornos, Han-Zhong Feng, Bruno Rizzuti, Martina Palomino-Schätzlein, David Wieczorek, José L. Neira, J.-P. Jin

**Affiliations:** 1 IDIBE, Universidad Miguel Hernández, Alicante, Spain; 2 Department of Physiology, Wayne State University School of Medicine, Detroit, Michigan, USA; 3 CNR-NANOTEC, Licryl-UOS Cosenza and CEMIF.Cal, Department of Physics, University of Calabria, Cosenza, Italy; 4 Department of NMR, Centro de Investigación Príncipe Felipe Valencia, Spain; 5 Department of Molecular Genetics, Biochemistry, and Microbiology, University of Cincinnati College of Medicine, Cinncinnnati, Ohio, USA; 6 Instituto de Biocomputación y Física de Sistemas Complejos, Zaragoza, Spain

**Keywords:** troponin I, muscle contractility, peptide conformation, computer modeling, skinned cardiac muscle, hypertrophic cardiomyopathy, αTm, α-tropomyosin, CD, circular dichroism, COSY, correlation spectroscopy, CSP, chemical shift perturbation, DOSY, diffusion ordered spectroscopy, DQF, double quantum filtered, HcTnI-C27, C-terminal 27-mer peptide of human cardiac TnI, HcTnI-C27-H, Arg192His mutant of the C-terminal 27-mer peptide of human cardiac TnI, HFpEF, heart failure with preserved ejection fraction, ITC, isothermal titration calorimetry, mAb, monoclonal antibody, MD, molecular dynamics, MW, molecular weight, NMR, nuclear magnetic resonance, NOE, nuclear Overhauser effect, NOESY, nuclear Overhauser effect spectroscopy, RMSD, root mean square deviation, ROE, rotating-frame Overhauser enhancement, ROESY, rotating-frame Overhauser enhancement spectroscopy, pCa50, pCa required for 50% maximum activation of myofibrils, SL, sarcomere length, TFE, 2,2,2-trifluoroethanol, Tm, tropomyosin, *T*_m_, thermal denaturation midpoint, Tn, troponin, TnI, troponin I, TOCSY, total correlation spectroscopy, TPPI, time proportional phase incrementation, tr-NOESY, transferred NOESY, TSP, sodium trimethylsilyl [2,2,3,3-^2^H_4_] propionate, UV, ultraviolet, WT, wild-type

## Abstract

The conserved C-terminal end segment of troponin I (TnI) plays a critical role in regulating muscle relaxation. This function is retained in the isolated C-terminal 27 amino acid peptide (residues 184–210) of human cardiac TnI (HcTnI-C27): When added to skinned muscle fibers, HcTnI-C27 reduces the Ca^2+^-sensitivity of activated myofibrils and facilitates relaxation without decreasing the maximum force production. However, the underlying mechanism of HcTnI-C27 function is unknown. We studied the conformational preferences of HcTnI-C27 and a myopathic mutant, Arg192His, (HcTnI-C27-H). Both peptides were mainly disordered in aqueous solution with a nascent helix involving residues from Trp191 to Ile195, as shown by NMR analysis and molecular dynamics simulations. The population of nascent helix was smaller in HcTnI-C27-H than in HcTnI-C27, as shown by circular dichroism (CD) titrations. Fluorescence and isothermal titration calorimetry (ITC) showed that both peptides bound tropomyosin (αTm), with a detectably higher affinity (∼10 μM) of HcTnI-C27 than that of HcTnI-C27-H (∼15 μM), consistent with an impaired Ca^2+^-desensitization effect of the mutant peptide on skinned muscle strips. Upon binding to αTm, HcTnI-C27 acquired a weakly stable helix-like conformation involving residues near Trp191, as shown by transferred nuclear Overhauser effect spectroscopy and hydrogen/deuterium exchange experiments. With the potent Ca^2+^-desensitization effect of HcTnI-C27 on skinned cardiac muscle from a mouse model of hypertrophic cardiomyopathy, the data support that the C-terminal end domain of TnI can function as an isolated peptide with the intrinsic capacity of binding tropomyosin, providing a promising therapeutic approach to selectively improve diastolic function of the heart.

The contraction and relaxation of striated muscles, *i.e.*, cardiac and skeletal muscles, are regulated by Ca^2+^ *via* the troponin complex in sarcomeric myofilaments. Troponin consists of three protein subunits: the Ca^2+^-binding subunit troponin C (TnC), the inhibitory subunit troponin I (TnI), and the tropomyosin (Tm)-binding subunit troponin T (TnT) (1, 2, 3, 4). Muscle contraction results from a rise of cytosolic Ca^2+^ that binds TnC to induce allosteric changes in TnC, TnI, TnT, and Tm, which allows the myosin heads to form strong cross-bridges with actin filament to activate myosin ATPase and generate power strokes and the shortening of sarcomeres (5).

Downstream of the Ca^2+^-receptor TnC, TnI acts as an allosteric signal transducer to regulate myofilament activity critical to muscle relaxation. Vertebrate TnI has diverged into three muscle-type isoforms (3) with largely conserved structures where the C-terminal end segment is the most conserved with a very high degree of sequence similarity among muscle type isoforms and across species (6) (Fig. 1). X-ray crystallography studies determined the folded structure of partial troponin complex containing whole TnC, most part of TnI, and a small portion of TnT (7, 8). The C-terminal end 27 residues of TnI (C27) were not resolved in the crystal structures, indicating a high mobility.

**Figure 1:**
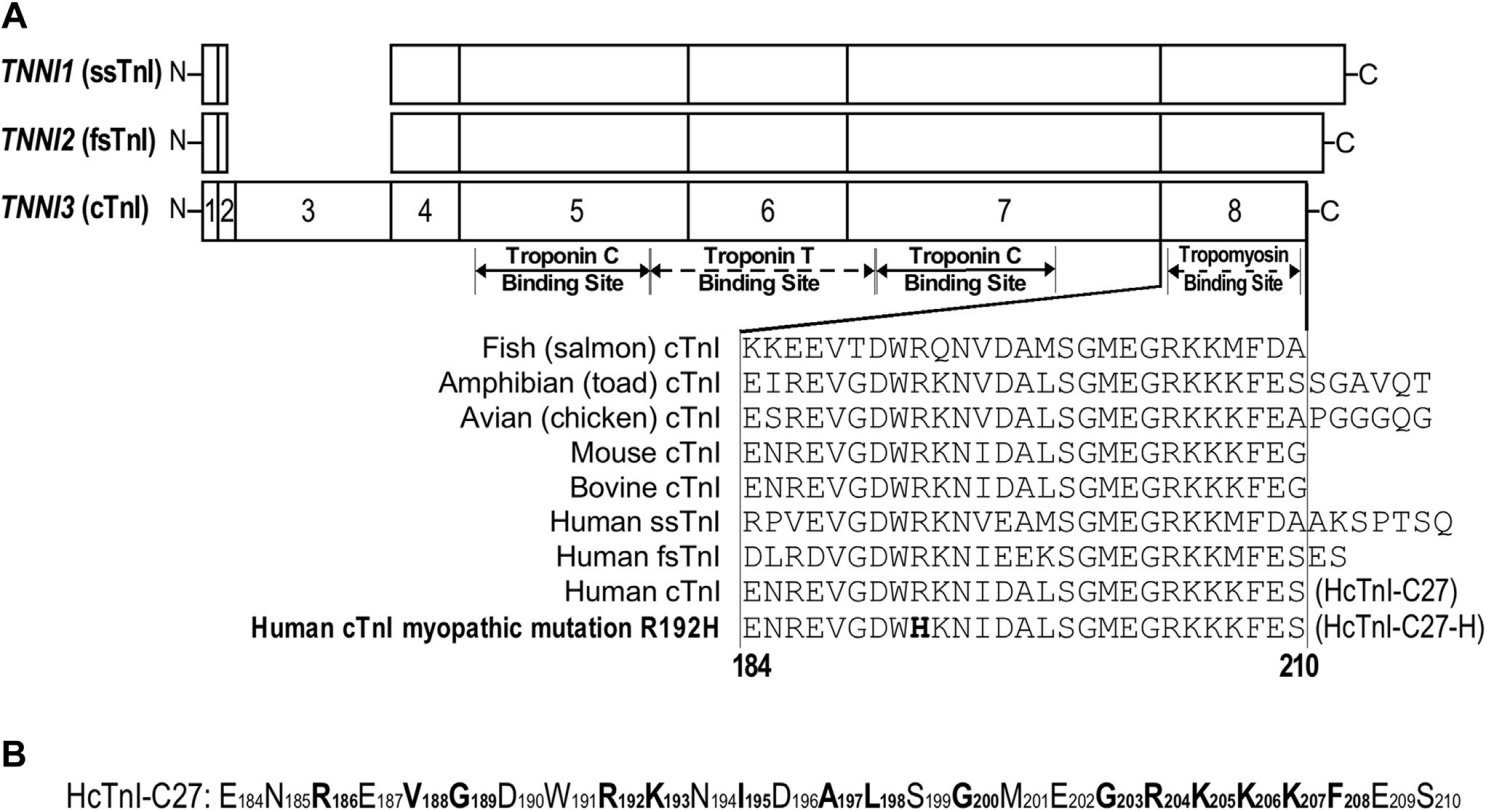
**The highly conserved C-terminal peptide of TnI.***A*, linear structures of the three isoforms of human TnI are aligned to show their exon organizations, major functional sites, and the location of the mAb TnI-1 epitope. The regions resolved in the crystal structure of human cardiac and chicken fast skeletal muscle troponin are shaded. The highly conserved amino acid sequences of the exon 8-encoded C-terminal end segment of cardiac (cTnI), slow skeletal muscle, and fast skeletal muscle isoforms from representative vertebrate species are shown by the alignment. The restrictive cardiomyopathy mutation, Arg192His, is shown in bold. *B*, the amino acid sequence of HcTnI-C27 is outlined with the residues numbered according to their position in intact human cardiac TnI. The residues in bold are those labeled with ^13^C and ^15^N in this study.

The C27 segment of cardiac TnI is critical to cardiac function (9). Mutation in this segment impairs cardiac muscle relaxation (10). We have previously shown that it is a Ca^2+^-regulated exposed allosteric structure in reconstituted troponin complex and binds tropomyosin at low Ca^2+^-state, indicating a role in the inhibitory function of TnI during muscle relaxation (11). Independent of the TnI backbone, isolated C27 peptide folds into a native conformation recognized by an anti-TnI C-terminus monoclonal antibody (mAb) TnI-1 (6) in physiological solution and retains binding affinity for Tm (12). The mAb TnI-1 recognized epitope in the C-terminal end peptide of TnI is conserved in all vertebrate TnI sequenced to date. A restrictive cardiomyopathy mutation (Arg192His) in the C-terminal end segment of cardiac TnI (13) diminishes the bindings to mAb TnI-1 and Tm (12).

When added to skinned cardiac muscle strips, the C27 peptide of human cardiac TnI (HcTnI-C27) causes reduction of myofilament Ca^2+^-sensitivity especially at the activated state without decreasing the maximum force production (12). The data indicate that the C-terminal end segment of TnI is a regulatory element of troponin, which retains the native configuration and physiological effect on myofilament Ca^2+^-desensitization in the form of free peptide. Without negative inotropic impact, this short peptide may be developed into a novel reagent to selectively facilitate the relaxation of activated cardiac muscle for the treatment of diastolic dysfunction and heart failure.

To establish the underlying mechanism, it is necessary to understand how free HcTnI-C27 peptide folds into functional structure. In the present study, we examined the conformational preferences of isolated HcTnI-C27 peptide in solution and the restrictive cardiomyopathic mutant HcTnI-C27-H (Arg192His) by using a combination of experimental and MD simulation approaches. The results demonstrate that both peptides populated a nascent-helix conformation, of which the amount of helical conformation was reduced in the mutant peptide that exhibits impaired physiological effect on Ca^2+^-sensitivity and contractility of cardiac muscle. HcTnI-C27 binds αTm with low-micromolar affinity, acquiring a helix-like conformation. With HcTnI-C27’s potent Ca^2+^-desensitization effect on skinned cardiac muscle from a mouse model of hypertrophic cardiomyopathy, the data support that the C-terminal end domain of TnI can function in the form of isolated peptide to modify muscle contractility as a potential therapeutic reagent to improve diastolic function for the treatment of heart failure.

## Results

### HcTnI-C27 and HcTnI-C27-H peptides are monomers in physiological buffer and populate a nascent helix

We first determined the tendency of HcTnI-C27 and HcTnI-C27-H peptides to self-associate by measuring the translational diffusion coefficient, *D*. Both peptides showed predicted *R*_h_ values comparable with those theoretically calculated for random-coil peptides (14) with the same molecular weight, suggesting that both peptides were monomeric and mainly disordered in the physiological buffer condition at the concentration tested (100 μM) (Table 1).

**Table 1:** Hydrodynamic properties of the C27 peptides (at pH 7.2 and 10 °C)

Peptide	*D* (cm^2^ s^−1^) × 10^6^ (*R*_h_, Å)^a^	*R*_h_, Å^b^	Molecular weight (Da)
HcTnI-C27	9.9 ± 0.1 (13.3 ± 0.3)	15 ± 4	3180.55
HcTnI-C27-H	10.1 ± 0.3 (13.0 ± 0.4)	15 ± 4	3161.50

a Errors are uncertainties to Equation 2. The *R*_h_ values were obtained by comparison with the *R*_h_ of dioxane (2.12 Å).

b Calculated from the scale law: *R*_h_ = (0.027 ± 0.01) MW^(0.50 ± 0.01)^ (14).

**Figure 2:**
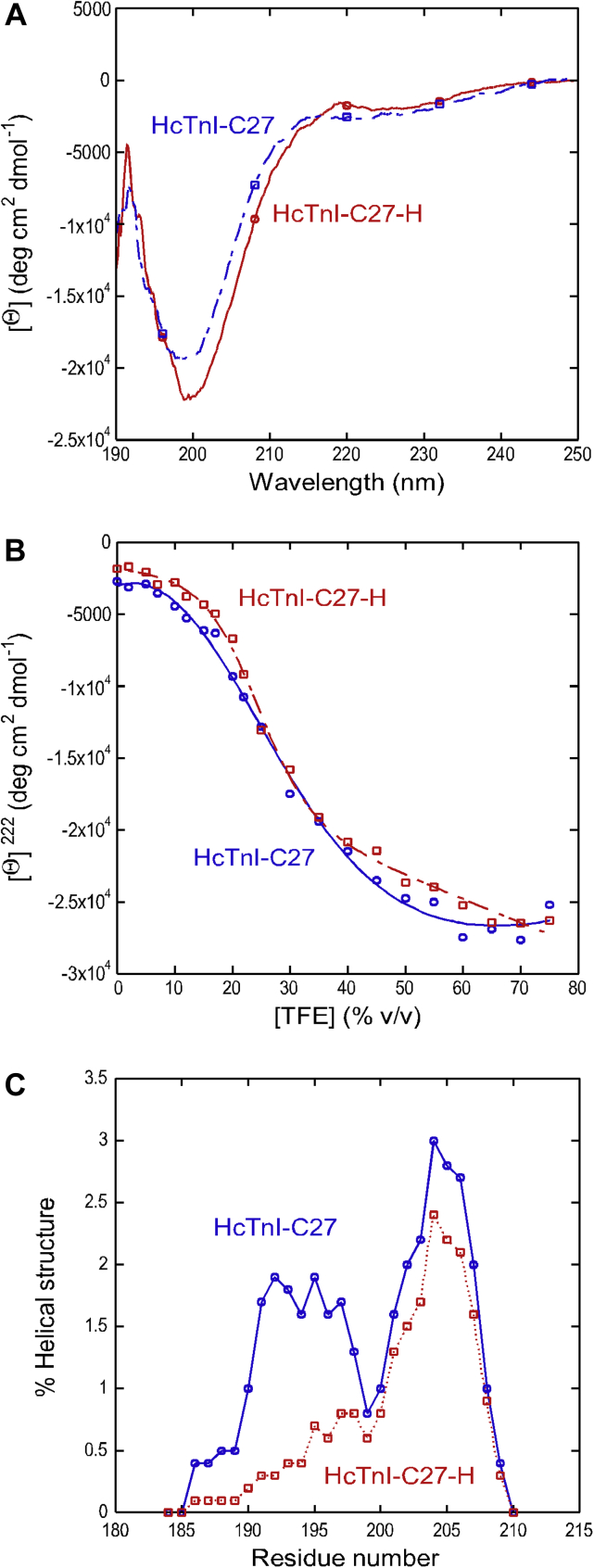
**CD spectra of HcTnI-C27 and HcTnI-C27-H peptides.***A*, far-UV CD spectra at pH 7.2 and 5 °C. *B*, the sigmoidal change in the [Θ] at 222 nm, [Θ]^222^, as the TFE concentration was increased. The line is the fitting to a two-state model (20, 21). *C*, AGADIR (22, 23, 24, 25, 26, 27) helical percentages for both peptides.

The far-UV CD spectra of HcTnI-C27 and HcTnI-C27-H peptides in aqueous solution both showed an intense band at 200 nm with a small shoulder at 222 nm, suggesting that they were mainly disordered (Fig. 2*A*). The presence of the 222 nm shoulder could be due to a population of turn- or helix-like structures and/or the existence of aromatic residues in the peptides (Trp191 and Phe208 in HcTnI-C27; and Trp191, His192, and Phe208 in HcTnI-C27-H), which also absorb at 222 nm (15, 16). While the possible interference of those residues is considered, the amount of helical structure in both peptides was estimated from the CD spectra by using two methods as follows.

First, from the molar ellipticity at 222 nm, [Θ]^222^, we estimated the percentage of helical structure for the peptides assuming that the value of [Θ]^222^ for a fully formed helix is −39,500 deg cm^2^ dmol^−1^ (17). The estimated values were 6.7% (−2669.8 deg cm^2^ dmol^−1^) for HcTnI-C27 and 4.6% (−1829.7 deg cm^2^ dmol^−1^) for HcTnI-C27-H (Fig. 2*A*). Spectral deconvolution using the DICHROWEB web page (18, 19) yielded a prediction of a very small population of helical structure in both peptides: 0.5% to 10%, depending on the algorithm used, for HcTnI-C27, and 0.4% to 6% for HcTnI-C27-H. Considering the possible interference of aromatic residues indicated above, these differences in the deconvolution of the spectra of both peptides, or in the helix estimation from the ellipticity at 222 nm, are taken with caution. And second, TFE titrations followed by CD were employed to estimate the equilibrium constant (*K*) for the disordered ↔ helical reaction (20, 21), assuming that there were no intermediates (as suggested by the presence of an isodichroic wavelength for each peptide titration). Unlike the above analyses, which make assumptions about how the ellipticity signal at 222 nm relates to secondary structure content, TFE titration allows the determination of helical content derived from the free-energy change of such equilibrium. The [Θ]^222^ increased in absolute value for both peptides as TFE concentration was raised (Fig. 2*B*). The titration for HcTnI-C27 was more cooperative than that for HcTnI-C27-H, with *m*-values of 42 ± 14 kcal/(mol %) and 117 ± 20 kcal/(mol %), respectively. However, the [TFE]_1/2_ (the midpoint of the transition in % (v/v)) did not differ significantly: 16 ± 8% for wild-type and 23 ± 2% for the mutant (Fig. 2*B*). These values yielded a 7.5% of helical population for HcTnI-C27 (*K* = 0.0818) and 1% for the mutant (*K* = 0.012). These findings suggested that the tendency to populate an α-helical structure was larger in the wild-type peptide.

Additionally, we used AGADIR (22, 23, 24, 25, 26, 27) to have a theoretical estimate of the helical population of both peptides. AGADIR is widely used to qualitatively predict the helical behavior of peptides by CD, mimicking residue-level helical tendency of peptides in NMR (24, 25, 26, 27). The predictions (at 10 °C and pH 7.2) yielded very small percentages of helical structure (Fig. 2*C*) for both peptides: 1.43% for HcTnI-C27 and 0.82% for HcTnI-C27-H. It is important to note that these values indicate that the percentage of helical structure in both peptides was small as anticipated for short 27-residue-long peptides. However, the change of only one amino acid in the mutant produced notable difference in helicity (Fig. 2*C*).

Therefore, the far-UV CD data indicate that both peptides weakly populated helix-like conformations, and such population was slightly higher in the wild-type peptide than in the myopathic mutant.

### Amino acid residues involved in the nascent helix of HcTnI-C27 and HcTnI-C27-H peptides

**Figure 3:**
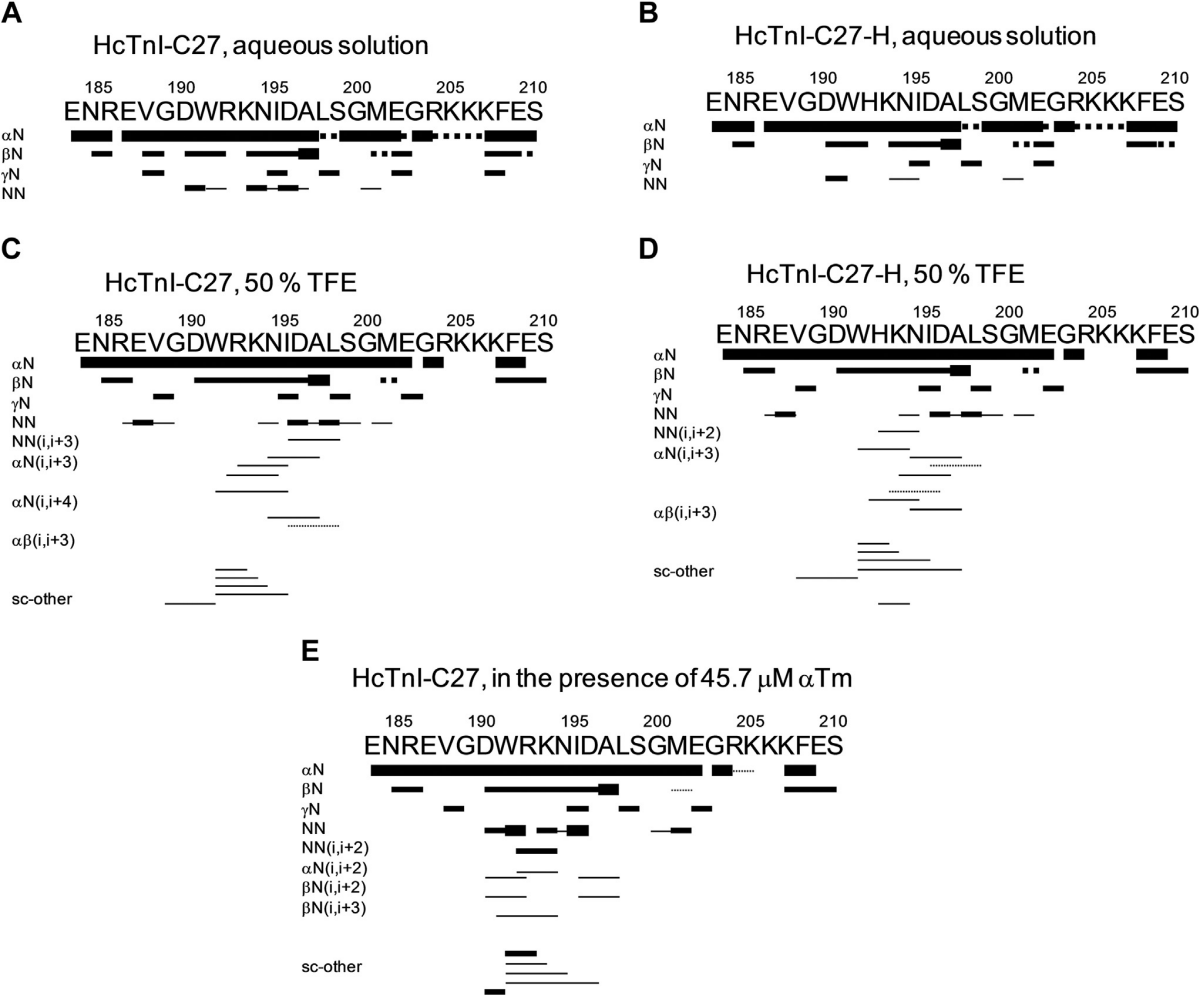
**NMR structure of HcTnI-C27 and HcTnI-C27-H peptides**. Summary of NMR data for the peptides under different conditions is shown. NOEs are classified into strong, medium, or weak according to the height of the bar underneath the sequence. The contacts were measured in the ROESY experiment. The *dotted lines* indicate NOE contacts, which could not be unambiguously assigned due to signal overlapping. The residue numbering corresponds to that of the whole sequence of human cardiac TnI. The symbols αN, βN, γN, and NN represent the sequential contacts between the corresponding protons of adjacent residues.

We carried out homonuclear 2D-^1^H-NMR experiments to determine the presence of the helix-like conformations. The peptides were mainly disordered in aqueous solution, but there were residual conformations as shown by two pieces of evidence. First, no long- or medium-range NOEs were detected (Fig. 3), but sequential NN(*i*, *i* + 1) NOEs (and ROEs) were observed for residues around Trp191 and Ile195 in the HcTnI-C27 peptide (Fig. 3*A*), with most of these NOEs not being present in HcTnI-C27-H (Fig. 3*B*). These contacts indicate the presence of a nascent helix, although it was not very stable, as shown by the absence of protected amide protons of the peptides in the presence of D_2_O (hydrogen/exchange experiments) (Fig. S1). And second, the sequence-corrected conformational shifts (28, 29, 30) (Δδ) of H_α_ protons in the polypeptide patch Asp190-Arg192 were outside the commonly accepted range for random-coil peptides (|Δδ| ≤ 0.1 ppm) (Tables S1 and S3). The ^13^C chemical shifts of the C_α_ and C_β_ of the residues labeled (*i.e.*, Arg186, Val188, Gly189, Arg192, Lys193, Ile195, Ala197, Leu198, Gly200, Gly203, Arg204, Lys205, Lys206, Lys207, and Phe208) were not very different from those reported for random-coil polypeptide chains (30) (Table S3).

We performed NMR experiments in the presence of 50% TFE, when the titration of both peptides had reached a plateau (Fig. 2*B*). Both the NOE pattern (presence of NN(*i*, *i* + 1), NN(*i*, *i* + 2), NN(*i*, *i* + 3), αN(*i*, *i* + 3), αN(*i*, *i* + 4), αβ(*i*, *i* + 3) contacts) (Fig. 3, *C*–*D*) and the conformational shifts of the H_α_ protons (Tables S2 and S4) indicate the presence of helix-like conformations between Trp191 and Gly200. This polypeptide patch involves the same region acquiring an α-helix (α5 helix) in the whole TnI-TnC-TnT2 complex (PDB accession number 1VDI) (31). It is important to note that the organic solvent does not induce helical structure, but it only enhances the population of this structure in those regions with an intrinsic tendency to acquire such type of conformation.

To investigate whether different pH yielded a different NOE pattern in NMR experiments for the helicities of the two peptides, we also carried out experiments at pH 4.5 (Tables ST6 and ST7) for both peptides to test experimentally the qualitative predictions of AGADIR (22, 23, 24, 25, 26, 27), which suggested a lower percentage of helical structure at this pH due to global electrostatic effects in the peptides: 1.00% for the wild-type peptide and 0.70% for the myopathic mutant. As seen at pH 7.0 described above, we observed that in HcTnI-C27 the sequential amide–amide NOEs between Val188-Gly189, Asp190-Trp191, Lys193-Asn194, Ile195-Asp196, Met201-Glu202, and Gly203-Arg204 were detected. On the other hand, for HcTnI-C27-H, only the amide–amide NOEs between Asn194-Ile195, Met201-Glu202, and Gly203-Arg204 were observed. Thus, the number of sequential NOEs, characteristic of a nascent helix, in both peptides at pH 4.5 was smaller than at physiological pH (Fig. 3).

**Figure 4:**
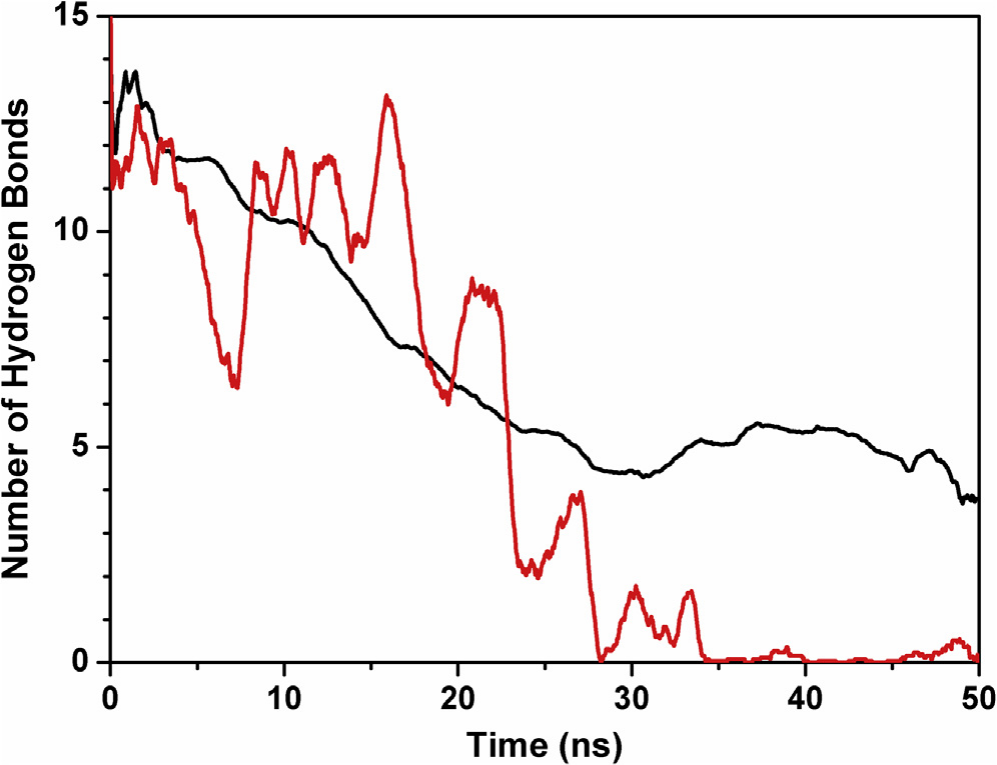
**Simulated secondary structure of HcTnI-C27 and HcTnI-C27-H peptides**. Number of hydrogen bonds forming α-helical structure obtained in MD study is plotted as a function of time for HcTnI-C27 (*black*) and HcTnI-C27-H (*red*). For clarity, data are averaged over a 100-point time window.

**Figure 5:**
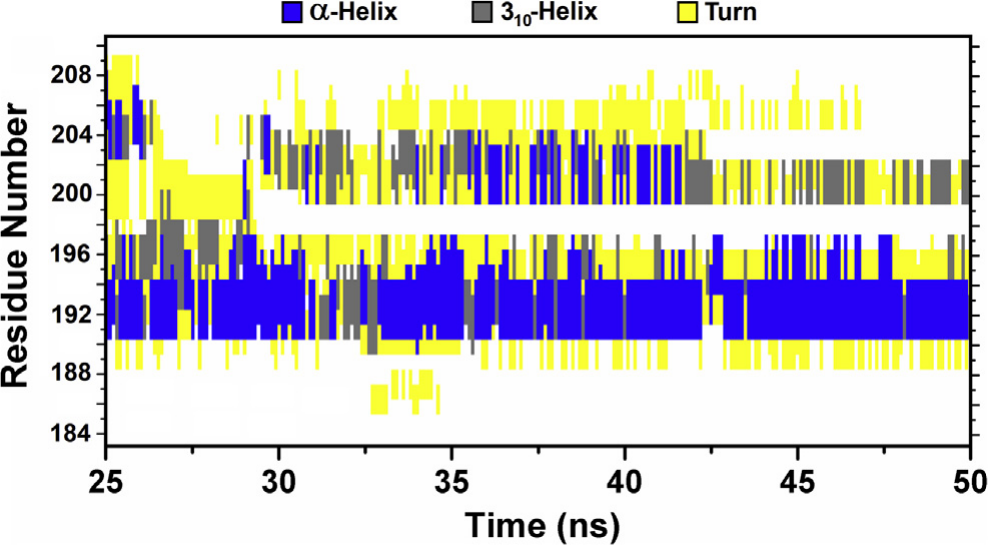
**Residues with secondary structure in HcTnI-C27 peptide**. Details of the secondary structure obtained in simulation in the time window of 25 to 50 ns are shown as that when the number of hydrogen bonds involved in α-helical structures has reached a plateau (see Fig. 4). A persistent presence of α-helix is visible starting from residue Trp191.

To further confirm the propensity of the peptides to populate helical conformations, we carried out MD simulations for 50 ns in explicit solvent using the water model TIP3P (32). The starting structures were built on the basis of the TnI-TnC-TnT2 complex (31). Figure 4 shows that in the structure of the wild-type peptide HcTnI-C27, the number of hydrogen bonds forming α-helical structure decreased from the initial number (about 13–15 hydrogen bonds), and after 25 ns equilibrated to a final value of 5. In contrast, the HcTnI-C27-H peptide was less stable and lost all its structure before the end of the simulation. This finding is a direct indication of a decreased helical propensity due to the Arg192His mutation (in agreement with the far-UV CD results, the TFE titration, the deconvolution of far-UV CD spectra, and the NMR results). Figure 5 reports in detail the type of secondary structure of the wild-type peptide obtained at equilibrium as a function of the residues. The results show that a residual α-helix was present starting at the Trp residue and extended up to the following four amino acid residues along the sequence.

The NMR and MD data conclude that, at residue level, isolated HcTnI-C27 had native helix-like structure in aqueous solution around Trp191 and a tendency to extend it into the following few residues. On the other hand, HcTnI-C27-H had a smaller population of helix-like structures and, thus, this mutation disrupted (although not fully) the structure around the aromatic moiety.

### Binding of HcTnI-C27 and HcTnI-C27-H peptides to tropomyosin

**Figure 6:**
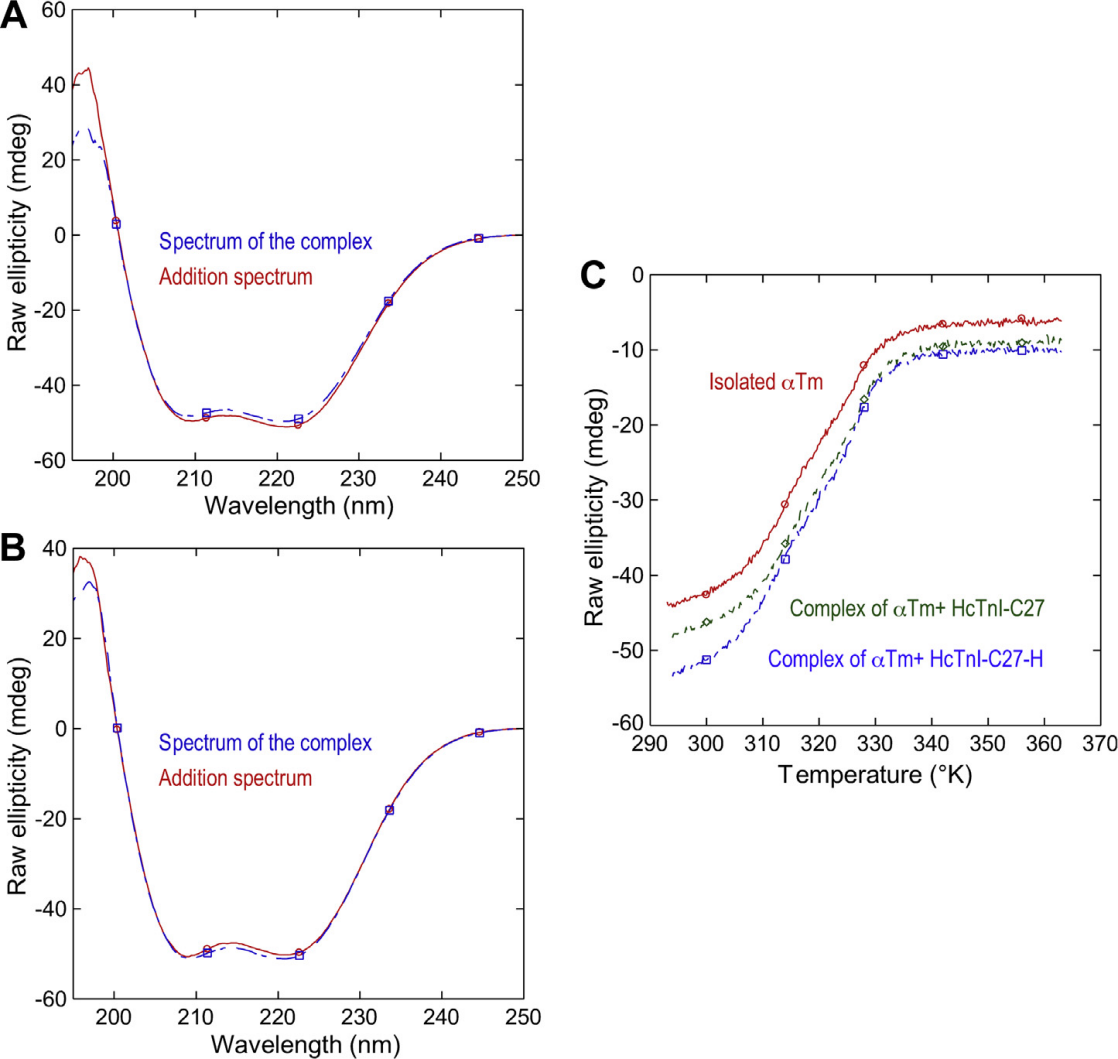
**Binding of HcTnI-C27 and HcTnI-C27-H peptides to αTm shown in far-UV CD spectra.***A*, far-UV CD spectra of the equimolar complex formed by HcTnI-C27 and αTm and that obtained by the addition of the spectra of both isolated molecules. *B*, far-UV CD spectra of the equimolar complex formed by HcTnI-C27-H and αTm and that obtained by the addition of the spectra of both isolated molecules. *C*, thermal denaturation monitored by ellipticity changes at 222 nm for isolated αTm, and its complexes with HcTnI-C27 and HcTnI-C27-H peptides.

We further tested the binding of HcTnI-C27 and HcTnI-C27-H peptides to Tm, a potential target of TnI’s inhibitory regulation of the activation and deactivation of myofilaments (11, 12). First, we monitored the binding by using far-UV CD measurements for the formation of complex of equimolar amounts of the corresponding peptide and protomer of tropomyosin. HcTnI-C27 showed small changes in the addition spectrum (obtained by the sum of the spectra of isolated αTm and HcTnI-C27) and that of the complex (Fig. 6*A*). On the other hand, HcTnI-C27-H showed changes similar to those of the wild-type peptide (Fig. 6*B*), but the variations were in the opposite direction. We explored the binding between αTm and the peptides in thermal-denaturation experiments followed by CD. If such binding occurs for both peptides, the thermal denaturation midpoint of the potential complexes should increase when compared with that of αTm alone. Figure 6*C* shows that is indeed the case. In the presence of peptides, the midpoint of the thermal denaturation of αTm increased from 43.1 °C ± 0.3 deg. C (316.25 ± 0.3 K) to 46.7 °C ± 0.2 deg. C (319.85 ± 0.2 K) for HcTnI-C27 and 47.7 °C ± 0.4 deg. C (320.25 ± 0.4 K) for HcTnI-C27-H. Therefore, the results show unambiguously that both peptides bound αTm and that upon binding there were changes in the structure of the peptides and/or tropomyosin.

**Figure 7:**
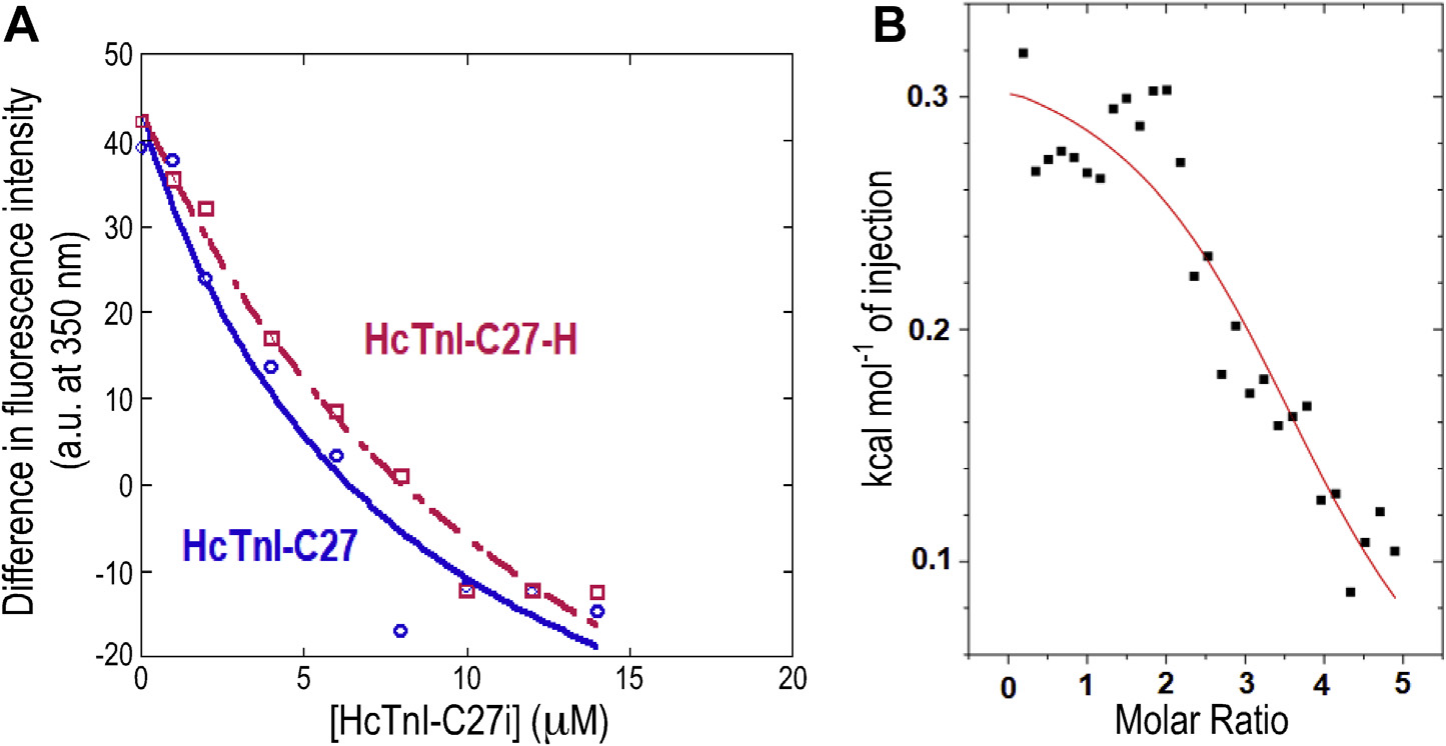
**Binding of HcTnI-C27 and HcTnI-C27-H peptides to αTm shown in fluorescence and ITC assays.***A*, titration fluorescence intensities at 350 nm obtained by excitation at 280 nm after subtraction of the intensity of the spectrum of the isolated peptide at the corresponding concentration. The line through the experimental points is the fitting to Equation 1. *B*, the isotherm from calorimetric titrations of αTm with HcTnI-C27 in phosphate buffer, pH 7.0, 25 °C shows the enthalpy change upon binding as a function of the peptide to protein molar ratio. The line is the fitting to a 1:1 binding equation. The raw data are provided in Supplementary Materials.

Although peptide binding affinity and protein structural stabilization are intimately related, there is no direct correlation (that is, peptides exhibiting the same affinity do not necessarily induce the same stabilization effect). Therefore, protein stability increments are not useful to rank peptide binding affinities, and the increased stability observed upon thermal-denaturation may be the result of unspecific interactions between the peptides and Tm. To quantitatively measure the binding between both peptides and tropomyosin, we carried out titration fluorescence measurements and ITC experiments. The fluorescence results yielded a *K*_d_ of 8 ± 4 μM and 15 ± 5 μM for HcTnI-C27 and HcTnI-C27-H mutant peptides, respectively (Fig. 7*A*). On the other hand, the ITC results (Fig. 7*B*) showed that the binding of wild-type peptide to αTm was characterized by a very small enthalpy change (0.35 ± 0.02 kcal/mol), which precluded the determination of the dissociation constant with high precision (raw data of the ITC for the wild-type peptide are shown in Fig. S2). Nevertheless, the estimated *K*_d_ value obtained from ITC for the wild-type peptide (11 ± 4 μM) is in line with the ones obtained from fluorescence. Accordingly, the binding of HcTnI-C27 to αTm is entropically driven, suggesting that the release of water molecules upon binding and structure gain (see below) may play a key role in the energetics of the molecular recognition process. We could not measure the affinity of HcTnI-C27-H by ITC because the curves were noisy and they did not yield any reliable *K*_d_. From the fluorescence data, we can also conclude that the affinity of the myopathic mutant peptide for αTm was lower than that of the wild-type peptide. It is important to note that the differences in the values of the dissociation constants obtained by fluorescence were small. However, it is evident that (i) the ITC only yielded a reliable value for the wild-type peptide; and (ii) this value was similar to that measured by fluorescence analysis, supporting that there were small, but distinct differences in the binding of the two peptides to αTm. On the other hand, the fact the HcTnI-C27-H induced a larger variation in the thermal denaturation midpoint, *T*_m_, of the thermal denaturation of the complex compared with that of isolated αTm could imply a higher affinity. The change in *T*_m_ induced by a ligand (peptide) on a protein depends on: (i) the affinity of the interaction; (ii) ligand and protein concentration; (iii) the enthalpy of the binding reaction; and (iv) the heat capacity of such reaction. The last two thermodynamic parameters are important because they govern how the affinity of the complex changes with the temperature (the van’t Hoff equation) and our thermal denaturation study is probing the thermal unfolding of the corresponding complex. If a ligand has an endothermic enthalpy in a binding reaction, when the temperature increases, the affinity of the complex rises until the enthalpy (positive because it is endothermic) gets null, and then (assuming the heat capacity of the binding reaction is negative) from that temperature on, the affinity decreases because the enthalpy is becoming negative (exothermic). On the other hand, if the ligand has an exothermic enthalpy, when we increase the temperature, the affinity decreases upon raising the temperature. The different value of the enthalpy and the different heat capacity for the wild-type and the myopathic peptide result in a value of the *T*_m_ larger for the complex of the latter.

We then addressed the question whether the structure acquired by HcTnI-C27 in the presence of αTm was the same as that acquired by the mobile C-terminal domain of TnI in the TnI-TnC-TnT complex (31) by using transferred NOESY (tr-NOESY) (Fig. 3*E*) in the presence of submicromolar concentrations of αTm (Table S5 and Fig. S3). The presence of NN(*i*, *i* + 1), NN(*i*, *i* + 2), αN(*i*, *i* + 2), βN(*i*, *i* + 2), βN (*i*, *i* + 3) contacts indicates that HcTnI-C27 was adopting a helix-like structure around Trp192, encompassing similar residues as in the α5 helix of the mobile domain of the TnI-TnC-TnT_2_ (31); the number of NN(*i*, *i* + 1) contacts was very similar to those measured in isolated peptide in aqueous solution, but their intensity was higher (Fig. S3); furthermore, the stability of the helix was not very high, as there were no protected amide protons in D_2_O for the peptides (Fig. S1). We tried to obtain a structure, by using CYANA software (33), with the 79 NOEs measured in tr-NOESY for the wild-type peptide, and none of them involving residues more than five amino acids apart. Such amount of NOEs for a peptide of this length is very small, and it probably yields a structure that is not reliable. In fact, the 20 obtained structures by CYANA had a very low target function (mean of 0.23), but they showed a high root mean square deviation (RMSD) of 4.12 ± 0.76 (for all backbone atoms from Arg186 to Lys207), and the percentage of residues in the most favored regions of the Ramachandran plot was very low (16.7%), although there were no residues in the disallowed regions.

The binding was mapped by using relaxation measurements in the absence or presence of αTm at a protomer concentration five times of that of the peptide. It is important to indicate that upon addition of such amount of αTm, we did not observe any large variation in proton chemical shifts of the cross-peaks for most of the labeled residues (*i.e.*, Arg186, Val188, Gly189, Arg192, Lys193, Ile195, Ala197, Leu198, Gly200, Gly203, Lys205, Lys206, and Phe208), but we only noticed changes in chemical shifts for Arg204 and Lys207 near the C terminus of the peptide (as shown by the chemical shift perturbation (CSP), Fig. S5). However, we did observe a broadening of all cross-peaks in the presence of αTm (Fig. S4). This broadening resulted in a quantitatively measured increase of the *R*_2_ rates (see below) and it indicates that the dynamics of the peptide (in the nanosecond-to-millisecond time regime) is modified by the presence of αTm. In the absence of tropomyosin, the peptides move faster showing smaller *R*_2_ values, indicating interactions between the peptide and αTm in solution and different conformational exchanges. It is important to note at this stage that we were not able to measure properly the intensity of Arg204 in the ^1^H-^15^N {NOE} spectrum of the bound peptide although we were able to measure its *R*_1_ and *R*_2_ rates, which were 2.3 ± 0.3 s^−1^ and 5.1 ± 0.7 s^−1^, respectively. The values of the *R*_1_ rates for all residues were not significantly modified in the presence of αTm (Fig. S6*A*) and remained similar (a mean of 2.32 ± 0.22 s^−1^ for the bound peptide; and a mean of 2.17 ± 0.14 s^−1^ for the isolated peptide). On the other hand, the *R*_2_ rates of all residues increased in the presence of αTm, indicating binding between the two molecules. Although the resonances of Arg192 and Lys193 did not show any change in chemical shifts, they showed large variations in the *R*_2_ rates (Fig. S6*B*). In addition, the NOE values also showed the largest variations for Arg192, Lys193, Ile195, and Ala197, presenting values larger in the free peptide than in the αTm-bound form (Fig. S6*C*).

**Figure 8:**
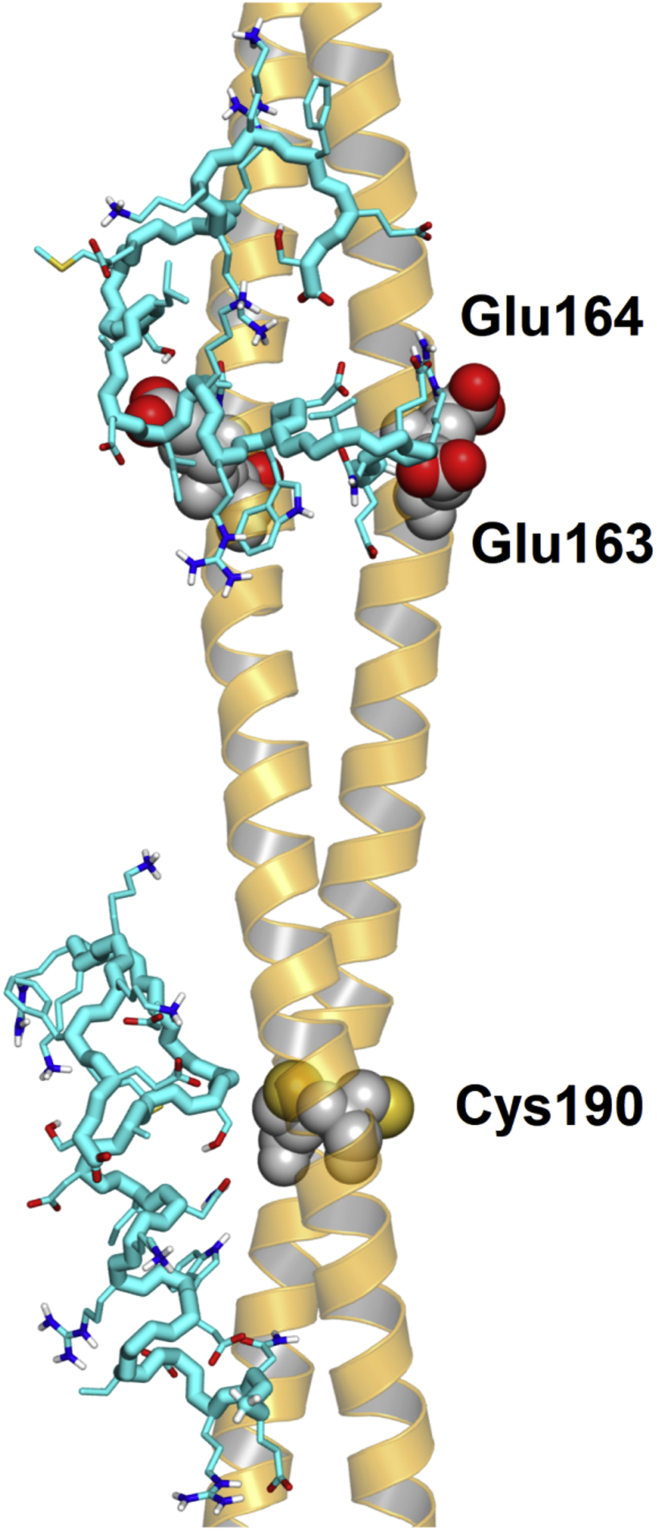
**Docking of HcTnI-C27 peptide on tropomyosin.** Representative conformations of the wild-type peptide in the two predicted binding locations of tropomyosin are shown with protein residues explicitly labeled. For clarity, the peptide main chain (cyan) is represented omitting the O and H atoms, whereas the side chains have atoms in standard colors.

Molecular docking was performed to predict the most favorable binding location of HcTnI-C27 peptide on the surface of Tm dimer. Due to the exceedingly large number of degrees of freedom in the peptide structure, a crude approximation was used that consisted of allowing rotations only around its main chain φ and ψ dihedral angles and with the exclusion of those with secondary structure maintained in the MD simulations (*i.e.*, around Trp191). The results indicated that the most favorable docking poses (with predicted binding energy of about −6.5 kcal/mol) clustered around two distinct locations (Fig. 8). One was in correspondence of residue Cys190 of tropomyosin, which is a site that has been documented for physiological importance, whereas the other was in between the two consecutive acidic residues Glu163 and Glu164. In both cases, Trp191 of HcTnI-C27 appeared to be a key residue in the binding due to its ability to form hydrophobic interactions with Tm with an additional effect on the side chain of Ile195 in the subsequent turn of the α-helix. This modeling result supports the findings of fluorescence analysis, where we followed the changes in tryptophan fluorescence to map the binding to Tm (Fig. 7*A*). The modeling study suggests a possible rationale for the stabilization of the helical structure in the binding site, which would explain the further extension of the helical conformation up to residue Leu198, as indicated by the tr-NOESY results (Fig. 3*E*). Electrostatic interactions also played a role in the binding of HcTnI-C27 and Tm, especially in the second location identified, due to the charge–charge attraction with the small acidic patch present on the protein surface. Such interactions would be partially disrupted by the Arg192His mutation in the HcTnI-C27-H peptide. These long-range electrostatic interactions could explain the change in the chemical shifts of the cross-peaks of positively charged Arg204 and Lys207 (see above) (Fig. S4) in the presence of a fivefold (protomer) amount of αTm, although they are located far away from Trp191.

### Impaired effect of the myopathic mutant peptide HcTnI-C27-H on myofibril contractility

**Figure 9:**
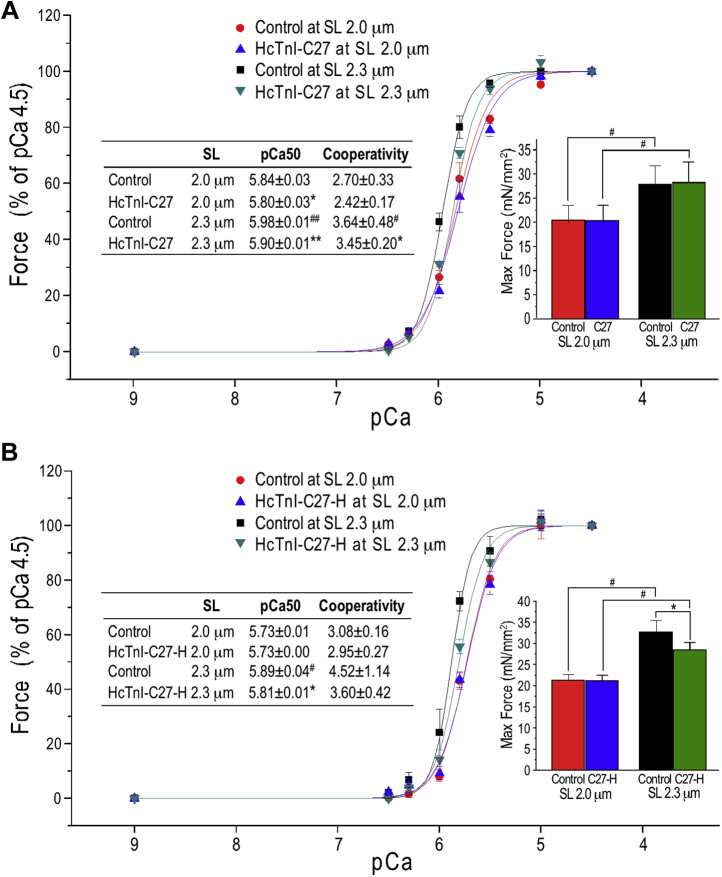
**HcTnI-C27 reduces Ca**^**2+**^**sensitivity of skinned mouse cardiac muscle, whereas HcTnI-C27 has a weaker effect.***A*, force–pCa curves show that the treatment with HcTnI-C27 decreased Ca^2+^ sensitivity of skinned mouse ventricular papillary muscle with larger effect at sarcomere lengths (SL) of 2.3 μm than that at SL 2.0 μm. The maximum force production was not affected (the inset bar graph). *B*, HcTnI-C27-H mutant peptide had less effect at SL of 2.3 μm and no effect at SL of 2.0 μm. The maximum force production was not affected at SL 2.0 μm but significantly decreased by the adding of HcTnI-C27-H mutant peptide at SL of 2.3 μm (the inset bar graph). Ca50, Ca^2+^ concentration for 50% maximum force. Values are presented as mean ± SE. N = 5 in HcTnI-C27 studies and N = 6 in HcTnI-C27-H studies. Statistical analysis was done using one-way repeated measures ANOVA following with pairwise comparison. ∗*p* < 0.05 and ∗∗*p* < 0.01 HcTnI-C27 peptide treatment *versus* control; ^#^*p* < 0.05 and ^##^*p* < 0.01 SL 2.3 μm *versus* SL 2.0 μm.

To establish the functional significance of the structural data, contractility studies showed that the addition of HcTnI-C27 peptide reproduced the Ca^2+^-desensitization effect with a right shift of the force-pCa curve in skinned mouse cardiac muscle strips (Fig. 9*A*) as reported previously (12). The effect was greater at longer sarcomere length of 2.3 μm than that at sarcomere length of 2.0 μm (Fig. 9*A*). In comparison, the HcTnI-C27-H mutant peptide had no Ca^2+^-desensitization effect at sarcomere length of 2.0 μm and less effect at sarcomere length of 2.3 μm (Fig. 9*B*). Whereas HcTnI-C27 peptide plausibly does not affect maximum force (Fig. 9*A*), HcTnI-27-H significantly decreased maximum force production, indicating an impaired function possibly underlying the cardiomyopathic phenotype of the Arg192His mutation in cardiac TnI (13).

### HcTnI-C27 has potent Ca^2+^-desensitization effect on skinned cardiac muscle of transgenic mouse containing a hypertrophic cardiomyopathy mutation in cardiac αTm

**Figure 10:**
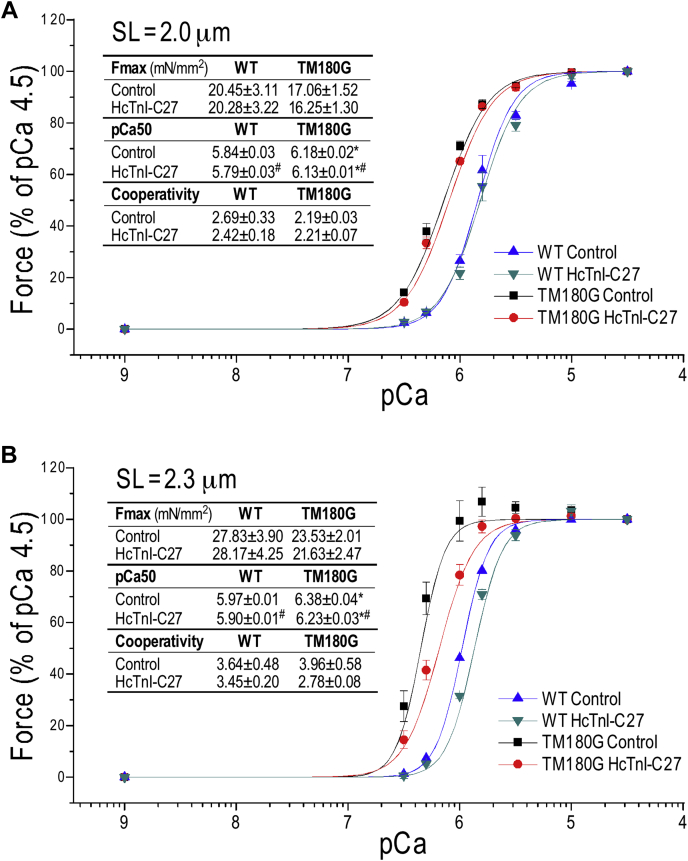
**Therapeutic effect of HcTnI-C27 on alleviating the Ca**^**2+**^**hypersensitivity of skinned papillary muscle strips of Tm180 G mice.** Force–pCa relationship was studied at sarcomere length (SL) of 2.0 μm (*A*) and 2.3 μm (*B*) for the effect of 20 μM HcTnI-C27. The maximal force development, pCa50 (the Ca^2+^ concentration at 50% of the maximum force at pCa 4.5), and Hill fitting of data normalized to maximum force for the cooperativity of Ca^2+^-activation are shown in the inset tables. Tm180G hypertrophic failing cardiac muscle with higher Ca^2+^-sensitivity (higher pCa50) than wild-type (WT) controls at SL of 2.0 μm and 2.3 μm. HcTnI-C27 treatment decreased Ca^2+^-sensitivity of Tm180G cardiac muscle significantly more than that of WT control especially at SL of 2.3 μm. The Ca^2+^-desensitization effect of HcTnI-C27 peptide is more potent at higher [Ca^2+^] corresponding to the activated state and does not decrease the maximal force development. Values are presented in Mean ± SE. N = 6 fibers in WT and n = 3 in Tm180G. ∗*p* < 0.05 *versus* WT in unpaired Student’s *t*-test; ^#^*p* < 0.05 *versus* control in paired Student’s *t*-test.

Force-pCa studies showed that skinned sections from transgenic mouse left ventricular papillary muscle expressing a hypertrophic cardiomyopathy mutation E180G in cardiac αTm (Tm180G) (34) had the anticipated higher Ca^2+^ sensitivity than that of wild-type cardiac muscle (Fig. 10). At sarcomere length 2.0 μm, the treatment of 20 μM HcTnI-C27 had a notably larger Ca^2+^-desensitization effect on Tm180G cardiac muscle than that on wild-type muscle (Fig. 10*A*). At sarcomere length 2.3 μm, HcTnI-C27 had much higher Ca^2+^-desensitization effects on Tm180G cardiac muscle (Fig. 10*B*). With the significant myofilament Ca^2+^-desensitization effect especially at higher [Ca^2+^] corresponding to activated state, HcTnI-C27 did not decrease maximum force development of the failing Tm180G cardiac muscle. The results demonstrate an appealing therapeutic function of HcTnI-C27 peptide for the treatment of hypertrophic cardiomyopathies and heart failure with diastolic dysfunction.

## Discussion

TnI is the inhibitory subunit of troponin and plays a critical role in regulating muscle contraction and relaxation (3, 35). We recently reported that free peptide of the highly evolutionarily conserved C-terminal 27-residue-long end segment of TnI (C27, Fig. 1*A*) retains a native epitopic structure in solution and the endogenous binding affinity for tropomyosin (12). Adding free HcTnI-C27 peptide to skinned cardiac muscle strips produced an activated state-specific Ca^2+^ desensitization effect, demonstrating a novel mechanism to improve muscle relaxation (12). Cardiac muscle dysfunction, especially diastolic dysfunction, and heart failure are a worldwide medical challenge that lacks effective treatment. Myocardial contractility is essential for heart function and an ultimate target for the treatment of heart failure. The diastolic function of cardiac muscle determines the efficacy of ventricular filling and is critical to cardiac pumping based on the Frank–Starling mechanism. More that 50% of clinical heart failure cases are due to diastolic dysfunction that currently has no specific treatment (36). Derived from the inhibitory subunit of troponin, the HcTnI-C27 peptide is a promising therapeutic reagent of physiological function and safe endogenous origin. To investigate the molecular basis of the effect of HcTnI-C27 peptide on myofilament function for the treatment of diastolic heart failure, the results of our present study provide the following mechanistic insights.

### Free C27 peptide populates a nascent helix in aqueous solution

The first result of our far-UV CD and NMR studies is that both HcTnI-C27 and HcTnI-C27-H peptides populated helical structures around Trp191. This population was smaller in HcTnI-C27-H containing the Arg192His mutation that causes diastolic dysfunction of the heart (13). The simulation results further suggest that the helical structure is labile, with disordered states prone to form transient hydrogen bonds.

The helical population seemed to be triggered by the presence of Trp191, as larger number of sequential NN(*i*, *i* + 1) NOEs were clustered around those residues (Fig. 3), consistent with the observation that tryptophan residues are involved in triggering helical structure or hydrophobic clusters in free peptides (37, 38, 39). Our MD study also indicated that the helical propensity of the Trp residue extends to the four downstream amino acids along the sequence. It is worth noting that the presence of an arginine immediately after the tryptophan increases the population of the helical structure, as judged by the differences observed in far-UV CD and NMR (Figs. 2 and 3). The introduction of a bulky side chain close to the indole moiety as that of the imidazole ring of the Arg192His mutation in HcTnI-C27-H probably hampers backbone hydrogen-bond formation and promotes helix disruption. However, we cannot rule out the importance of electrostatic interactions in the triggering of a helical fold in the isolated peptides among the side chains of either His192 or Arg192 with those of Asp190 or Asp196 (the closest residues) or other amino acids located farther away; in fact, the NMR spectra of both peptides at pH 4.5 showed basically the same chemical shifts for most of the protons, except for those belonging to Glu or Asp residues in the polypeptide chain (Tables ST6 and ST7), which are titrating in that pH interval. In addition, the presence of an arginine close to the tryptophan could allow the formation of a hydrogen bond between both side chains, as it has been observed in X-ray crystallography structures of protein complexes (40). The Arg192His mutation in cardiac TnI causes severe diastolic functions of the heart (13), and the impaired helical propensity of HcTnI-C27-H peptide suggests a likely molecular mechanism underlying the pathogenesis.

### C27 peptide binds αTm with micromolar affinity

Our results show that HcTnI-C27 binds αTm with an affinity in the order of low micromolar (∼10 μM). This finding is in agreement with the previous report that the HcTnI-C27 peptide is capable of competing at low micromolar concentration with intact bovine cardiac TnI for binding to αTm, whereas the HcTnI-C27-H mutant peptide showed a decreased effect in the competitive binding assay (12). Supporting this notion, our fluorescence measurements in the present study show that the Tm-binding affinity of HcTnI-C27-H was lower than that of the wild-type peptide (Fig. 7*A*), and it did not result in a detectable amount of heat uptake during ITC protein binding experiment. We suggest that the decreased affinity of the mutant peptide was due to its smaller population of helical structure, and then, the entropy variation, Δ*S*, occurring upon binding was much larger for HcTnI-C27-H, decreasing the value of the Δ*G*. Therefore, a high population of nascent helix is critical to attain binding of αTm at physiological conditions. As a pathological consequence, the reduced Tm-binding affinity of the Arg192His mutation may impair the inhibitory function of TnI during the relaxation of the cardiac muscle to cause diastolic dysfunction and severe restrictive cardiomyopathy (13).

Upon binding to αTm, the structural dynamics of the peptide was altered (Fig. S6) due to complex formation. However, the conformation of the peptide was not altered in the presence of αTm, as shown by the absence of changes in chemical shifts in the HSQC spectra (Fig. S4). In addition, not all residues showed the same variations in the *R*_2_ and NOE values with the largest variations in chemical shifts located at the C terminus (Fig. S6), and a larger number of sequential NN(*i*, *i* + 1) NOEs located around Arg192 and Lys193 (Fig. 3). These findings indicate that those residues were affected by severe conformational exchange upon binding and were probably among the first to bind αTm in the encounter complex. Interestingly, the NOE values were higher in the isolated peptide than in the bound species, indicating that upon binding to Tm the peptide became more disordered in the nanosecond-picosecond time regime. This behavior (disorder upon binding), also known as cryptic disorder, has been described in proteins involved in signaling pathways (41, 42, 43, 44), where a particular region of the polypeptide chain changes its structure. To the best of our knowledge, our data report for the first time the cryptic disorder in an isolated peptide where the binding with a physiological partner protein induced substantial change in dynamics. This property suggests a conformational selection mechanism upon binding to αTm, favoring a specific conformation within the ensemble explored by the peptide with a higher flexibility at particular residues (Arg192, Lys193, Ile195, and Ala197) in the timescale of nanoseconds to picoseconds, whereas the dynamics of the rest of the residues was unchanged. Therefore, the HcTnI-C27 mutant is capable of tuning and modulating its sustained binding to αTm and function, by modifying the dynamics of particular residues involved in the nascent-helical structure.

While NMR spectra could not provide any indication of the anchoring region of HcTnI-C27 on Tm, the docking study indicated two potential sites with favorable binding affinity, in correspondence of residues Cys190 and of the small acidic patch Glu163-Glu164 in αTm. The docking technique was not capable of supplying direct information on the dynamics of the peptide, but it suggested a molecular conformation that favors the stabilization of a helix-like conformation based on the possibility of forming hydrophobic interactions driven by residues Trp191, Ile195, and Leu198 in HcTnI-C27. Hydrophobic surfaces are important in forming encounter complexes (45), or recently for the complex between a protein and a peptide (46). Accordingly, our docking findings suggest that the Arg192His mutation in HcTnI-C27-H peptide would disrupt the key binding region on the peptide surface, disfavoring the association with tropomyosin.

### The myopathic mutant peptide HcTnI-C27-H has decreased Ca^2+^ desensitization effect

The primary function TnI is the inhibitory regulation of muscle contraction, which is critical to muscle relaxation and diastolic function of the heart (3, 35). Heart failure with preserved ejection fraction (HFpEF) is a common clinical condition that has no specific treatment for (47). The myofilament Ca^2+^-desensitizing effect of HcTnI-C27 peptide is derived from the endogenous function of TnI and demonstrates an approach to selectively modulate contractile kinetics downstream of Ca^2+^ activation without reducing maximum force production (Fig. 9*A*). Supporting the plausible effect of HcTnI-C27 peptide on increasing diastolic function of cardiac muscle as a potential treatment of HFpEF, HcTnI-C27-H peptide containing the restrictive cardiomyopathy mutation Arg192His (10) shows a detectable decrease in the Ca^2+^-desensitizing effect but a significantly decreased maximum force production (Fig. 9*B*), indicating the decreased population of helical structure and absence of a folded conformation may underlay the cardiomyopathy phenotype.

### HcTnI-C27 peptide as a potent Ca^2+^-desensitizer for the treatment of diastolic dysfunction of cardiac muscle

The results that HcTnI-C27 peptide has much larger Ca^2+^-desensitization effect on Tm180 G mutant cardiac muscle than that on WT muscle especially at longer sarcomeres (Fig. 10) demonstrate a potent therapeutic function. E180 G mutation in cardiac αTm causes severe hypertrophic cardiomyopathy and heart failure with a feature of increased myofilament Ca^2+^ sensitivity (34). Similar to the effects on WT cardiac muscle (12), the Ca^2+^-desensitization effect of HcTnI-C27 is significantly higher at higher [Ca^2+^] corresponding to the activated state (Fig. 10*B*). This feature is plausible to facilitate myocardial relaxation without decreasing the maximum force development (Fig. 10). Therefore, HcTnI-C27 peptide provides a promising therapeutic reagent with high correcting effect on diseased myofilaments for the treatment of hypertrophic cardiomyopathies and diastolic heart failures.

Our comprehensive biophysical characterization of the C-terminal end peptide of TnI demonstrated that the C-terminal end domain of TnI can function as an isolated peptide that preserves the intrinsic capacity of binding Tm in myofilaments, with physiological effect on tuning muscle contractility and selectively improving diastolic function for use as a therapeutic reagent for the treatment of heart failure. The much less effect of HcTnI-C27 on WT (Fig. 9) than that on Tm180G cardiac muscle strips (Fig. 10) demonstrates a correcting effect that is safely restricted within physiological range. The data describe a structure and mechanistic foundation for the development of a new myofilament Ca^2+^ desensitizer to treat diastolic heart failures while minimizing side effects.

## Experimental procedures

### Animals

All animal procedures were carried out using protocols approved by the Institutional Animal Care and Use Committee of Wayne State University and were conducted in accordance with the Guiding Principles in the Care and Use of Animals, as approved by the Council of the American Physiological Society.

### Materials

Wild-type, HcTnI-C27, and Arg192His mutant, HcTnI-C27-H, peptides, comprising residues from Glu184 to Ser210 of human cardiac TnI (Fig. 1*A*), were synthesized using a commercial service as described (12) with a nominal purity of 98%, which was confirmed by mass spectrometry to be larger than 95%. Peptide concentrations were determined from UV absorbance at 280 nm (48) of the sole Trp191 present in both peptides.

^15^N,^13^C-labeled wild-type HcTnI-C27 peptide was produced with labeled residues at: Arg186, Val188, Gly189, Arg192, Lys193, Ile195, Ala197, Leu198, Gly200, Gly203, Arg204, Lys205, Lys206, Lys207, and Phe208 (Fig. 1*B*) by chemical synthesis at GenScript Biotech (New Jersey, USA) at a nominal purity of 98%, which was confirmed by mass spectrometry to be larger than 95%.

Rabbit cardiac α-tropomyosin (αTm) was purified from frozen hearts using conventional biochemical methods as described (12). The concentration of stock used in the present study was estimated by UV absorbance at 278 nm from the six Tyr residues in the sequence of the protomeric unit (48).

Deuterium oxide and 2,2,2-d_3_ trifluoroethanol (TFE) (96.5% purity) were obtained from Apollo Scientific (Stockport, UK). Sodium trimethylsilyl [2,2,3,3-^2^H_4_] propionate (TSP), deuterated acetic and acetate sodium salt, and 2,2,2- trifluoroethanol were from Sigma (St Louis, MO). All other chemicals used in the study were analytical grade. Deionized water was purified using a Millipore system.

### Circular dichroism (CD)

Far-UV CD spectra were collected on a Jasco J810 (Tokyo, Japan) spectropolarimeter interfaced with a Peltier unit. Far-UV measurements were performed with 20 μM of each peptide in 50 mM sodium phosphate buffer (pH 7.2) in 0.1 cm-pathlength quartz cells (Hellma, Belgium) at 5 °C. Experiments in CD and NMR (see below) were carried out at low temperature to increase the population of a possible folded structure in the peptides (according to Boltzmann distribution). The band width was 1 nm, the response time was 2 s, and the scan speed was 50 nm/min. Raw ellipticity was converted to molar ellipticity, [Θ], and the amount of helical populations was determined as described (49).

For experiments in the presence of TFE, the same set of parameters as in aqueous solution experiments was used. Cosolvent concentrations were indicated in v/v (%). The helical populations for each peptide in the absence of cosolvent were determined by assuming a two-state equilibrium: disordered ↔ helical (as suggested by the presence of isodichroic wavelengths during the titrations of both peptides) (20, 21).

For detecting peptide–protein binding using far-UV CD, we prepared samples of HcTnI-C27 or HcTnI-C27-H with αTm at an equimolar concentration (10 μM, in protomer units for αTm). The rest of the experimental setup was the same as described above.

Thermal-denaturation experiments for the αTm/peptide complexes or αTm control were performed at constant heating rates of 60 °C/h and measured at a band width of 1 nm and response time of 8 s. Thermal scans were collected by following the changes in ellipticity at 222 nm from 20 to 90 °C. The apparent thermal-denaturation midpoint was estimated from a two-state equilibrium equation as described (49).

### Fluorescence spectroscopy

Fluorescence spectra were collected on a Cary Varian spectrofluorimeter (Agilent, USA), interfaced with a Peltier unit. Experiments were carried out in a physiological protein binding buffer containing 100 mM KCl, 3 mM MgCl_2_, 10 mM sodium phosphate, pH 7.0. The samples were prepared the day before and stored overnight at 5 °C and equilibrated at 25 °C for 1 h before the experiments. A 1 cm-pathlength quartz cell (Hellma) was used.

Titrations of the binding between HcTnI-C27 or HcTnI-C27-H peptide and αTm were performed with increasing concentration of the peptides (0–15 μM) and a fixed concentration of αTm (10 μM protomer). Using excitation at 280 or 295 nm with excitation and emission slits of 5 nm, the experiments were carried out at 25 °C. The dissociation constant of αTm/HcTnI-C27 or αTm/HcTnI-C27-H complexes, *K*_d_, was calculated by fitting the changes observed to the following equation (50, 51):

(1) $$F\;=\;F_{0}\;+\;\frac{\Delta F_{\max}}{2{\left[\alpha Tm\right]}_{T}}\left[\begin{array}{l} \left({\left[\alpha Tm\right]}_{T}+{\left[\text{HcTnI}-\text{C}27\text{i}\right]}_{T}+K_{d}\right)-\\ {\left(\begin{array}{l} {\left({\left[\alpha Tm\right]}_{T}+{\left[\text{HcTnI}-\text{C}27\text{i}\right]}_{T}+K_{d}\right)}^{2}\\ -4{\left[\alpha Tm\right]}_{T}{\left[\text{HcTnI}-\text{C}27\text{i}\right]}_{T} \end{array}\right)}^{1/2} \end{array}\right]$$

where *F* is the measured fluorescence at any particular concentration of the peptides after subtraction of the blank signal; Δ*F*_max_ is the maximal change in the fluorescence of the peptides when the whole amount of αTm is forming the complex; *F*_0_ is the fluorescence intensity when no peptide was added; [αTm]_T_ is the constant total concentration of αTm (10 μM); and [HcTnI-C27i]_T_ is that of the corresponding peptides, which is varied during the titration. The titration was repeated twice for each peptide. The absorbance of the corresponding peptide was kept lower than 0.1 units (at 280 nm) to avoid inner-filter effect during fluorescence excitation (52). Fitting Equation 1 to the data was carried out using KaleidaGraph (Synergy software, Reading, PA, USA).

### NMR

NMR experiments for the assignment (at two pH values and under different solvent conditions) and translational diffusion measurements of the peptides were performed at 10 °C on a Bruker Avance DRX-500 (11.7 T) spectrometer (Karlsruhe, Germany), equipped with a triple resonance probe and z-pulse field gradients. The transferred NOESY (with a mixing time of 300 ms) and the relaxation measurements were acquired in an Avance-II 600 MHz spectrometer (14.1 T) equipped with a triple resonance cryo-probe and z-pulse field gradients at 10 °C. Temperature of both probes was calibrated with methanol (53). All experiments with the peptides in aqueous solution or in the presence of 50% TFE were carried out at pH 7.2 in 50 mM sodium phosphate buffer, except those acquired at pH 4.5 to test for electrostatic effects in the isolated peptides, which were acquired in 50 mM deuterated acetate buffer. All spectra were referenced to external trimethylsilylpropanoic acid (TSP), by taking into account the pH-dependence of its signals (53). All samples, either in aqueous or TFE solutions, contained 10% D_2_O to lock the sample.

#### Translational diffusion NMR experiments (DOSY)

The working concentrations for both HcTnI-C27 and HcTnI-C27-H peptides were 100 μM. In each experiment, 64 scans were acquired. Translational self-diffusion measurements were performed with the pulsed-gradient spin-echo sequence in 100% D_2_O. The following relationship exists between the translational diffusion coefficient, *D*, and the delays used during the acquisition (49):

(2) $$\frac{I}{I_{0}}\;=\;-\exp\left(D\gamma_{H}^{2}\delta^{2}G^{2}\left(\Delta -\frac{\delta }{3}-\frac{\tau }{2}\right)\right)$$

where *I* is the measured peak intensity of the methyl resonances at a gradient strength; *I*_0_ is the maximum peak intensity of the methyl groups at the smallest gradient strength; *D* is the translational self-diffusion constant; δ is the duration of the gradient (2.25 ms); *G* is the gradient strength; Δ is the time (200 ms) between the gradients; γ_H_ is the gyromagnetic constant of the proton; and τ is the recovery delay between the bipolar gradients (100 μs). The gradient strength was varied in 16 lineal steps between 2% and 95% of the total power of the gradient coil of the probe. Data were plotted as *I*/*I*_0_ *versus G*^2^, and the exponential factor of the resulting curve is $D\gamma_{H}^{2}\delta^{2}\left(\Delta -\frac{\delta }{3}-\frac{\tau }{2}\right)$, from where *D* can be obtained. The gradient strength was calibrated by using the value of *D* for the residual proton water line in a sample containing 100% D_2_O in a 5-mm tube (49). In the samples containing both peptides, 1% of a final concentration of dioxane was added as an internal size marker; the hydrodynamic radius, *R*_h_, of each peptide was obtained by assuming that the *R* of dioxane is 2.12 Å (54). The DOSY experiment was repeated twice for each peptide. Data were fitted to Equation 2 by using KaleidaGraph.

#### 2D-^1^H-NMR homonuclear spectroscopy

Two-dimensional spectra with a spectral width of 6000 Hz in both dimensions were acquired in the phase-sensitive mode by using the time-proportional phase incrementation technique (TPPI) (55) for both peptides. Peptide concentrations were in the range 1.0 to 1.3 mM. At pH 7.2, standard DQF-COSY, TOCSY (80 ms), ROESY (250 and 300 ms), and NOESY (300 ms) experiments were performed; at pH 4.5, only NOESY and TOCSY (with the same mixing times as at pH 7.2) were acquired. Standard phase-cycling sequences were used to acquire the complete set of experiments to achieve assignments. The DQF-COSY experiment (56) was acquired with a data matrix size of 4096 × 512 (*t*_2_ and *t*_1_, respectively) and 1 s of recycle delay, 128 scans per *t*_1_ increment, and with the residual water signal attenuated by presaturation during the relaxation delay. TOCSY, ROESY, and NOESY experiments were acquired with a data matrix size of 4096 × 512 (*t*_2_ and *t*_1_, respectively); for the TOCSY, we used the MLEV17 spin-lock sequence (57), with a mixing time of 80 ms. Eighty scans were acquired per *t*_1_ increment, the residual water signal was removed by using the WATERGATE sequence (58). NOESY and ROESY spectra (59, 60) were collected with 128 scans per *t*_1_ increment in both types of experiments, with the residual water signal removed in both experiments by the WATERGATE sequence. Mixing times were 300 ms. Data were zero-filled, resolution-enhanced, baseline-corrected with phase-shifted sine bell (for DQF-COSY) or square sine-bell window functions (for TOCSY, NOESY and ROESY), optimized in each spectrum, and processed with the Bruker TopSpin 2.1 software. ^1^H NMR resonances were assigned by standard sequential assignment processes (28). The random-coil chemical shift values of H_α_ protons were obtained from tabulated data in model peptides, corrected by neighboring residue effects (29, 30).

We also acquired tr-NOESY with unlabeled HcTnI-C27 at 600 MHz, with the same experimental setup described above, but a spectral width of 7200 Hz, and in the presence of the binding buffer (100 mM KCl, 3 mM MgCl_2_, 10 mM sodium phosphate, pH 7.0). Peptide concentration was 1.1 mM and that of αTm was 45.7 μM (protomer).

#### ^1^H, ^15^N-HSQC spectra and triple resonance experiments

The HSQC spectra of double-labeled HcTnI-C27 were acquired as described (61) at 14.1 T with 40 μM free peptide or 20 μM of double-labeled peptide and 104 μM (protomer) of αTm in the binding buffer. Other experimental details are as described previously (61). The ^1^H carrier was at the water frequency, which was removed by using the WATERGATE sequence (58); the spectral width in this dimension was 9 ppm. The ^15^N carrier was set at 120 ppm, and the spectral width in this dimension was 30 ppm.

We define the chemical shift perturbation (CSP) of the signals in the HSQC spectra as the Euclidean distance (62): $CSP=\sqrt{{\left(\Delta \delta_{H}\right)}^{2}+{\left(0.14\Delta \delta_{N}\right)}^{2}}$ where Δδ_H_ is the variation in the chemical shifts of the amide proton between the values in the HSQC spectra of wild-type peptide in the presence and in the absence of αTm, and the Δδ_N_ is the corresponding difference for the ^15^N nucleus.

Standard triple resonance experiments (53) were acquired in a Bruker 500 MHz magnet, with a concentration of 1.1 mM, to allow determination of the ^15^N and ^13^C frequencies for the labeled residues.

#### Relaxation measurements

NMR relaxation data included the acquisition of ^15^N-*R*_1_, ^15^N-*R*_2_ and ^1^H-^15^N {NOE} experiments with the double-labeled HcTnI-C27. Two samples were prepared, one with a concentration of 40 μM of double-labeled HcTnI-C27 in binding buffer; and the second containing 20 μM of double-labeled peptide and 104 μM (protomer) of αTm in the same buffer. All the relaxation measurements were determined in an interleaved manner to ensure that the experimental conditions were the same for the different relaxation delays. The ^15^N-*R*_1_, ^15^N-*R*_2,_ and ^1^H-^15^N {NOE} experiments were acquired for both samples by using enhanced sensitivity, gradient pulse sequences (63). The *T*_1_ (=1/*R*_1_) was measured in an interleaved way with typically 7 inversion-recovery delays, varying from 10 to 550 ms; *T*_2_ (=1/*R*_2_) was determined by collecting seven time points ranging from 10 to 400 ms. For the *T*_1_ and *T*_2_ pulse sequences, the delay between scans was 1.5 s. The ^1^H-^15^N {NOE} was measured by recording interleaved spectra in the absence or presence of proton saturation. The ^1^H-^15^N {NOE} spectrum recorded in the presence of proton saturation was acquired with a saturation time of 10 s. The ^1^H-^15^N {NOE} spectrum recorded without proton saturation incorporated a relaxation delay of 10 s. The ^1^H-^15^N {NOE} experiment (with and without saturation) was repeated twice for each sample. Spectra were recorded with 2048 × 180 complex matrices in the F_2_ and F_1_ dimensions, respectively, with typically 160 scans (NOE experiment) and 128 scans (*R*_1_ and *R*_2_ experiments) *per* F_1_ experiment. Spectral widths of 9 and 30 ppm were used for F_1_ and F_2_, respectively; the ^15^N carrier was set at 120 ppm and that of ^1^H was set on the water signal in all the experiments.

All the spectra were zero-filled in the F_1_ dimension (two to four times) and processed by using a shifted sine square window function in Topspin 2.1 software package (Bruker). The same window function was used throughout all the *T*_1_ and *T*_2_ experiments. Intensities of the cross-peaks were measured with Topspin 2.1. The *R*_1_ and *R*_2_ values were determined by fitting the measured peak-intensities to a two-parameter function: *I*(*t*) = *I*_0_exp (−*tR*_1,2_), where *I*(*t*) is the peak intensity after a relaxation delay *t*, and *I*_0_ is the intensity at time zero; uncertainties in the relaxation rates were error fittings from the above equation. The experimental data were fitted to such equation by using Kaleidagraph. The steady-state ^1^H-^15^N {NOE} values were determined from the ratios of the peak intensities with and without proton saturation (*i.e.*, NOE = *I*_sat_/*I*_nonsat_). The uncertainties of the NOE values were determined from the measured background noise levels during repeated experiments.

#### 1D ^1^H-NMR spectra in D_2_O

1D spectra were acquired in D_2_O with peptide concentrations in the range 30 to 50 μM in isolation or in the presence of submicromolar amounts of αTm (the buffers were those described above for the acquisition of HSQC spectra). The isolated peptides (or in the presence of the protein) were dissolved in D_2_O and kept on ice, while the magnet was shimmed with a dummy sample. Experiments were acquired with the WATERGATE sequence at 10 °C in the Avance DRX-500 (11.7 T) spectrometer. A number of 512 scans was acquired with a total matrix size of 32 K. The spectra were processed with Bruker TopSpin 2.1 software by using an exponential window function and apodized until 64 K.

### Isothermal titration calorimetry (ITC)

ITC experiments were carried out at 25 °C using a VP-ITC instrument from Microcal (Northampton, USA). αTm solution was dialyzed at 4 °C for 24 h against three changes of 2 l of 10 mM sodium phosphate, pH 7.0, containing 100 mM KCl and 3 mM MgCl_2_. The ligands (HcTnI-C27 and HcTnI-C27-H peptides) were dissolved in the same buffer recovered from the last dialysis of αTm. The ITC experiments involved sequential injections of 10 μl of stock solutions of concentrated ligand (609 μM for HcTnI-C27, and 723 μM for HcTnI-C27-H) loaded in a syringe connected to the calorimetric cell (1.4 ml) that contained αTm at a concentration of 30 μM (protomer). To correct for the heat evolved due to the dilution effect of adding the ligand solution to the calorimetric cell, an independent experiment was performed in which the same ligand solution was injected into the calorimetric cell loaded with buffer. After correction for the heat of dilution, the isotherm was fitted to the binding model assuming the existence of a single set of binding sites, by using the software provided by the manufacturer.

### Bioinformatic calculations

AGADIR (www.crg.es) (22, 23) website was used to predict the helical percentage of both peptides. DICHROWEB website (www.dichroweb.cryst.bbk.ac.uk) (18, 19) was used to predict the percentage of helical structure in both peptides from their far-UV CD spectra in aqueous solution.

### Molecular simulations

All-atom MD simulations were performed using the GROMOS simulation suite (64) with the force field Amber ff99SB-ILDN (65). The structure of the two peptides was built using the software Visual Molecular Dynamics (66), starting from the solution structure of the potential actin-binding domain of TnI reported in the entry 1VDI (31) in the Protein Data Bank. The peptides were solvated using the water model TIP3P (32). A simulation box with the shape of a rhombic dodecahedron was used, and a minimum distance of 12 Å from the solute was guaranteed, resulting in ∼5300 water molecules added. A single Cl^−^ counterion was also added to obtain a neutral system for the sole HcTnI-C27 peptide. Simulations in the isobaric–isothermal ensemble were carried out for 50 ns with a sampling time of 1 ps. Details on the barostat/thermostat reference values and coupling times, modeling of electrostatic and van der Waals interactions, and other specifics on the annealing and equilibration procedures used were as reported earlier (67, 68). The analysis of the secondary structure of the peptides was performed with the DSSP algorithm (69) and a sampling time of 10 ps.

### Molecular docking

The structure of Tm was modeled on the basis of entry 1C1G in the Protein Data Bank (70). Molecular docking was performed using AutoDock Vina 1.1.2 (71) and with graphical interface AutoDock Tools 1.5.6 (72). The system was described at atomic detail with the exception of apolar hydrogens, which were subsumed into the carbon atom they were bound to. The docking experiments were carried out using a “divide and conquer” approach: the whole protein surface was mapped with a blind search performed on smaller volumes having each a size 90 Å × 90 Å × 55 Å and with a grid spacing of 1 Å. The HcTnI-C27 peptide possesses 122 rotatable dihedral angles, which is a number too large for any current computational resource. Therefore, we considered rotatable bonds only in the main chain φ and ψ angles of the peptide that did not show a stable secondary structure in the preceding MD simulation, leading to a total of 44 rotatable bonds. The search was performed using an exhaustiveness factor 16 times larger than the default value (73), extending linearly the elapsed time while increasing exponentially the probability of finding the most accurate energetic minima (71). The maximum number of binding modes generated and the energy range obtained were always largely below the maximum values allowed, *i.e.*, 100 poses and 5 kcal/mol, respectively. We verified that the binding scores obtained were reproducible within 0.2 to 0.3 kcal/mol, due to the randomness in the docking search algorithm.

### Force-pCa measurements in skinned cardiac muscle strips

Permeabilized left ventricular papillary muscles from wild-type and Tm180G (34) mice in C57B/L6 background at 3 to 4 months of age were prepared using cryosection for contractility studies (12, 74). Cardiac muscle strips 120 to 150 μm wide and 35 μm thick were mounted between two aluminum T-clips and transferred to a chamber of a thermo-controlled stage (802D, Aurora Scientific) at 6 to 8 °C in a relaxation buffer (*N*,*N*-bis(2-hydroxyethyl)-2-aminoethane sulfonic acid 40 mM, EGTA 10 mM, MgCl_2_ 6.86 mM, ATP 5.96 mM, dithiothreitol 1 mM, creatine phosphate 33 mM, creatine kinase 200 U/ml, K-propionate 3.28 mM, pH 7.0, plus protease inhibitor cocktail). The muscle preparation was connected to a force transducer (403A, Aurora Scientific) and a length controller (322TSPC, Aurora Scientific). The buffer was then switched to a skinning solution (relaxation buffer containing 1% Triton X-100) for 20 min. After a wash with relaxation buffer, the permeabilized muscle strip was placed in pCa 9.0 buffer, and the sarcomere length was measured through a digital camera attached to the microscope and adjusted to 2.0 μm and 2.3 μm. Calcium activated force was examined at pCa 6.5, 6.3, 6.0, 5.8, 5.5, 5.0, and 4.5 at 15 °C. HcTnI-C27 or HcTnI-C27-H peptide was then added at 20 μM and the force–pCa measurements were repeated. The force–pCa curves were plotted and fitted using Hill exponential equation for data analysis and statistical analysis was performed using paired Student’s *t*-test.

## Data availability

All data are contained within the article and available from the corresponding authors: José L Neira, email: jlneira@umh.es; J.-P. Jin, email: jjin@med.wayne.edu.

## Conflict of interest

The authors declare that they have no conflicts of interest with the contents of the article.
