# Supplementary material for: The muscle-relaxing C-terminal peptide from troponin I populates a nascent helix, facilitating binding to tropomyosin with a potent therapeutic effect
Source: J Biol Chem. 2021 Jan 7;296:100228. doi: 10.1074/jbc.RA120.016012 (PMC7948816; doi:10.1074/jbc.RA120.016012)
Supplement: Supplementary Tables and Figures [file mmc1.pdf]

# Supporting Information

## SUPPPORTING TABLES

Table ST1: Chemical shifts (δ, ppm from TSP) of mutant HcTnI-C27-H in aqueous solution (pH 7.2 (50 mM sodium phosphate buffer), 10 ºC)[a]

| | NH | $H_\alpha$ | $H_{\beta2}$ | $H_{\beta3}$ | $H_{\gamma2}$ | $H_{\gamma3}$ | $H_{\delta2}$ | $H_{\delta3}$ | $H_\varepsilon$ | $H_\zeta$ |
|---|---|---|---|---|---|---|---|---|---|---|
| Glu184 | | 4.08 (-0.19) | 2.12 | | 2.43 | | | | | |
| Asn185 | 9.03 | 4.73 (0.04) | 2.80 | | | | | | | |
| Arg186 | 8.64 | 4.27 (-0.05) | 1.81 | | 1.58 | | 3.15 | | 7.19 (NH) | |
| Glu187 | 8.51 | 4.30 (-0.03) | 2.07 | | 2.47 | | | | | |
| Val188 | 8.24 | 4.12 (0.01) | 2.04 | | 0.94 (Me) | | | | | |
| Gly189 | 8.51 | 3.88 (-0.04) | | | | | | | | |
| Asp190 | 8.35 | 4.66 (0.17) | 2.77 | | | | | | | |
| Trp191 | 8.04 | 4.52 (-0.11) | 3.21 | | | | 10.14 (NH); 7.23 (2H); 7.45 (7H); 7.08 (6H); 7.13 (5H); 7.49 (4H) | | | |
| His192 | 8.12 | 4.35 (-0.08) | 2.88 | 3.07 | | | 7.01 (C4H) | | 8.49 (C2H) | |
| Lys193 | 8.10 | 4.11 (-0.03) | 1.70 | | 1.38 | | | | | |
| Asn194 | 8.51 | 4.64 (-0.08) | 2.79 | | | | 7.68; 6.99 | | | |
| Ile195 | 8.20 | 4.08 (-0.05) | 1.84; 0.89 (Me) | | 1.39 | 1.15 | 0.89 (Me) | | | |
| Asp196 | 8.53 | 4.63 (0.06) | 2.79 | 2.82 | | | | | | |
| Ala197 | 8.28 | 4.27 (-0.03) | 1.41 (Me) | | | | | | | |

| | | | | | | | | |
|---|---|---|---|---|---|---|---|---|
| Leu198 | 8.20 | 4.33 (-0.05) | | 1.63 | | | 0.87 (Me) | |
| Ser199 | 8.10 | 4.32 (-0.14) | | 3.86 | | | | |
| Gly200 | 8.46 | 3.95 (-0.08) | | | | | | |
| Met201 | 8.24 | 4.43 (-0.07) | | 2.02 | | 2.51 | | |
| Glu202 | 8.49 | 4.29 (-0.01) | 1.94 | | 2.02 | 2.31 | | |
| Gly203 | 8.46 | 3.95 (-0.02) | | | | | | |
| Arg204 | 8.09 | 4.29 (-0.03) | | 1.79 | | 1.63 | 3.17 | 7.23 (NH) |
| Lys205 (*) | 8.34 | 4.25 (-0.04) | | 1.71 | | 1.41 | | |
| Lys206 (*) | 8.34 | 4.25 (0.04) | | 1.71 | | 1.41 | | |
| Lys207 (*) | 8.34 | 4.25 (0.01) | | 1.71 | | 1.41 | | |
| Phe208 | 8.38 | 4.59 (-0.06) | | 3.06 | | | 7.24 | |
| Glu209 | 8.26 | 4.38 (0.09) | 1.89 | | 2.12 | 2.36 | | |
| Ser210 | 8.20 | 4.32 (-0.06) | | 3.88 | | | | |

[a] The (*) indicates those residues whose resonances could not be unambiguously assigned. For the $H_\alpha$ proton column, the value within parenthesis is the conformational shift ($\delta_{res}$-$\delta_{rc}$). The random-coil values for the sequence were obtained from: https://spin.niddk.nih.gov/bax/nmrserver/Poulsen_rc_CS/).

Table ST2: Chemical shifts (δ, ppm from TSP) of mutant HcTnI-C27-H in 50 % TFE (pH 7.2 (50 mM sodium phosphate buffer), 10 ºC)[a]

| | NH | $H_\alpha$ | $H_{\beta2}$ | $H_{\beta3}$ | $H_{\gamma2}$ | $H_{\gamma3}$ | $H_{\delta2}$ | $H_{\delta3}$ | $H_\varepsilon$ | $H_\zeta$ |
|---|---|---|---|---|---|---|---|---|---|---|
| Glu184 | | 4.12 (-0.14) | 2.16 | | 2.54 | | | | | |
| Asn185 | 8.94 | 4.68 (-0.01) | 2.92 | | | | 7.67; 6.90 | | | |
| Arg186 | 8.67 | 4.34 (0.02) | 1.89 | | 1.70 | | 3.18 | | | |
| Glu187 | 8.41 | 4.36 (0.04) | 2.13 | | 2.47 | | | | | |
| Val188 | 8.01 | 4.09 (-0.02) | 2.15 | | 1.03 (Me) | | | | | |
| Gly189 | 8.31 | 3.93 (0.01) | | | | | | | | |
| Asp190 | 8.29 | 4.67 (0.18) | 2.87 | | | | | | | |
| Trp191 | 8.10 | 4.53 (-0.10) | 3.40; 3.44 | | | | 9.97 (NH); 7.29 (2H); 7.46 (7H); 7.21 (6H); 7.01 (5H); 7.49 (4H) | | | |
| His192 | 7.93 | 4.15 (-0.32) | 3.10 | | 1.30 | | 6.93 (C4H) | | 8.49 (C2H) | |
| Lys193 | 7.88 | 4.22 (0.08) | 1.99 | | 1.72 | | | | | |
| Asn194 | 8.15 | 4.61 (-0.11) | 2.88 | | | | 7.25; 6.83 | | | |
| Ile195 | 8.04 | 3.99 (-0.14) | 1.90 | | 1.25 | 1.49 | 0.93 (Me) | | | |
| Asp196 | 8.34 | 4.45 (-0.12) | 2.95 | | | | | | | |
| Ala197 | 8.14 | 4.22 (-0.08) | 1.55 (Me) | | | | | | | |
| Leu198 | 8.24 | 4.24 (-0.14) | 1.82 | | 1.50 | | 0.96 (Me) | | | |
| Ser199 | 8.44 | 4.50 (0.04) | 4.02 | | | | | | | |

| | | | | | | | | | |
|---|---|---|---|---|---|---|---|---|---|
| Gly200 | 8.31 | 3.91 (-0.12) | | | | | | | |
| Met201 | 8.53 | 4.22 (-0.28) | | 2.20 | | | 2.54 | | |
| Glu202 | 8.17 | 4.33 (0.03) | | 2.26 | | | 2.66 | | |
| Gly203 | | | | | | | | | |
| Arg204 | 7.98 | 3.98 (-0.34) | | 1.81 | | | 1.34 | | |
| Lys205 | | | | | | | | | |
| Lys206 | | | | | | | | | |
| Lys207 | | | | | | | | | |
| Phe208 | 8.09 | 4.62 (-0.03) | 3.08 | | 3.30 | | | | 7.34 |
| Glu209 | 8.02 | 4.47 (0.18) | | 2.08 | | 2.24 | | 2.51 | |
| Ser210 | 8.10 | 4.52 (0.12) | | 3.90 | | | | | |

[a] For the $H_\alpha$ proton column, the value within parenthesis is the conformational shift ($\delta_{res}$-$\delta_{rc}$). The random-coil values for the sequence were obtained from: https://spin.niddk.nih.gov/bax/nmrserver/Poulsen_rc_CS/).

Table ST3: Chemical shifts (δ, ppm from TSP) of wild-type HcTnI-C27 in aqueous solution (pH 7.2 (50 mM sodium phosphate buffer), 10 ºC)[a]

| | NH | $H_\alpha$ | $H_{\beta 2}$ | $H_{\beta 3}$ | $H_{\gamma 2}$ | $H_{\gamma 3}$ | $H_{\delta 2}$ | $H_{\delta 3}$ | $H_\varepsilon$ | $H_\zeta$ |
|---|---|---|---|---|---|---|---|---|---|---|
| Glu184 | | 4.05 (-0.22) | 2.15 | | 2.45 | | | | | |
| Asn185 | 9.03 | 4.71 (0.06) | 2.78 | | | | | | | |
| Arg186 | 8.63 | 4.27 (-0.06) | 1.75 | | 1.56 | | 3.13 | | | |
| | (122.69) | [55.72] | (30.41) | | | | | | | |
| Glu187 | 8.53 | 4.29 (-0.04) | 2.03 | | 2.47 | | | | | |
| Val188 | 8.21 | 4.14 (0.02) | 2.07 | | 0.96 (Me) | | | | | |
| | (120.50) | [62.15] | (32.1) | | | | | | | |
| Gly189 | 8.52 | 3.85 (-0.07) | | | | | | | | |
| | (111.57) | [45.20] | | | | | | | | |
| Asp190 | 8.39 | 4.68 (0.18) | 2.79 | | | | | | | |
| Trp191 | 8.01 | 4.51 (-0.16) | 3.29 | | | | 10.19 (NH); 7.29 (2H); 7.46 (7H); 7.16 (6H); 7.10 (5H); 7.52 (4H) | | | |
| Arg192 | 7.80 | 3.91 (-0.24) | 1.59; 1.41 | | 0.94 | | 2.96 | | | |
| | (121.25) | [56.93] | (29.62) | | | | | | | |
| Lys193 | 7.94 | 4.12 (0.02) | 1.72 | | 1.35 | | | | | |
| | (119.77) | [56.92] | (32.35) | | | | | | | |
| Asn194 | 8.29 | 4.68 (0.00) | 2.79 | | | | 7.66; 6.97 | | | |

| | | | | | | | |
|---|---|---|---|---|---|---|---|
| Ile195 | 8.07 | 4.05 (-0.09) | 1.86; 0.91 (Me) | | 1.18 | 1.37 | 0.89 (Me) |
| | (120.50) | [61.42] | (38.63) | | | | |
| Asp196 | 8.50 | 4.63 (0.08) | 2.79 | 2.86 | | | |
| Ala197 | 8.22 | 4.23 (-0.08) | 1.39 (Me) | | | | |
| | (123.70) | [53.05] | (18.6) | | | | |
| Leu198 | 8.16 | 4.35 (-0.03) | 1.66 | | | | 0.89 (Me) |
| | (119.38) | [54.72] | (41.91) | | | | |
| Ser199 | 8.08 | 4.32 (-0.14) | 3.85 | | | | |
| Gly200 | 8.44 | 3.97 (-0.06) | | | | | |
| | (110.47) | [45.82] | | | | | |
| Met201 | 8.23 | 4.43 (-0.07) | 2.05 | | 2.54 | 2.57 | |
| Glu202 | 8.48 | 4.30 (0.00) | 1.95 | 2.07 | 2.28 | 2.50 | |
| Gly203 | 8.44 | 3.97 (0.00) | | | | | |
| | (109.03) | [45.81] | | | | | |
| Arg204 | 8.08 | 4.30 (-0.02) | 1.79 | | 1.63 | | 3.19 |
| | (119.69) | [56.21] | (30.52) | | | | |
| Lys205 | 8.34 | 4.20 (-0.09) | 1.68 | | 1.30 | | |
| | (122.77) | [56.31] | (33.15) | | | | |
| Lys206 | 8.34 | 4.20 (-0.02) | 1.68 | | 1.30 | | |
| | (121.04) | [57.32] | (32.72) | | | | |

| | | | | | |
|---|---|---|---|---|---|
| Lys207 | 8.32 | 4.24 (0.00) | 1.72 | 1.39 | |
| | (121.75) | [56.52] | (32.35) | | |
| Phe208 | 8.35 | 4.59 (-0.07) | 3.06 | | 7.25 |
| | (122.00) | [57.23] | (39.3) | | |
| Glu209 | 8.25 | 4.39 (0.09) | 1.90 2.11 | 2.38 | |
| Ser210 | 8.18 | 4.32 (-0.06) | 3.80 | | |

[a] For the $H_\alpha$ proton column, the value within parenthesis is the conformational shift ($\delta_{res}$-$\delta_{rc}$). The values within parenthesis for the NH colum correspond to the chemical shifts for the $^{15}N$ and those for the $H_\beta$ colum correspond to the chemical shifts of the $^{13}C_\beta$; the values within brackets for the $H_\alpha$ column correspond to the chemical shift of the $^{13}C_\alpha$. The random-coil values of protons for the sequence were obtained from: https://spin.niddk.nih.gov/bax/nmrserver/Poulsen_rc_CS/).

Table ST4: Chemical shifts (δ, ppm from TSP) of wild-type HcTnI-C27 in 50 % TFE (pH 7.2 (50 mM sodium phosphate buffer), 10 ºC)[a]

| | NH | $H_{\alpha}$ | $H_{\beta 2}$ | $H_{\beta 3}$ | $H_{\gamma 2}$ | $H_{\gamma 3}$ | $H_{\delta 2}$ | $H_{\delta 3}$ | $H_{\varepsilon}$ | $H_{\zeta}$ |
|---|---|---|---|---|---|---|---|---|---|---|
| Glu184 | | 4.10 (-0.17) | 2.16 | | | | | | | |
| Asn185 | 8.92 | 4.74 (0.09) | 2.86 | | | | 7.67; 6.85 | | | |
| Arg186 | 8.66 | 4.28 (-0.05) | 1.85 | | 1.66 | | 3.19 | | | |
| Glu187 | 8.41 | 4.36 (0.04) | 2.13 | | 2.44 | | | | | |
| Val188 | 7.98 | 4.05 (-0.07) | 2.11 | | 1.03 (Me) | | | | | |
| Gly189 | 8.27 | 3.95 (0.03) | | | | | | | | |
| Asp190 | 8.26 | 4.65 (0.15) | 2.86 | | | | | | | |
| Trp191 | 8.09 | 4.62 (-0.05) | 3.45 | | | | 9.88 (NH); 7.36 (2H); 7.46 (7H); 7.21 (6H); 7.10 (5H); 7.56 (4H) | | | |
| Arg192 | 7.94 | 3.78 (-0.37) | 1.71 | | 1.30 | | | | | |
| Lys193 | 7.78 | 4.21 (0.07) | 1.94 | | 1.72 | | | | | |
| Asn194 | 7.98 | 4.66 (-0.02) | 2.82 | 2.95 | | | 7.17; 6.79 | | | |
| Ile195 | 7.78 | 4.12 (-0.01) | 1.96 | | 1.25 | 1.59 | | | | |
| Asp196 | 8.45 | 4.60 (0.03) | 2.83 | 3.01 | | | | | | |
| Ala197 | 8.13 | 4.21 (-0.10) | 1.59 (Me) | | | | | | | |
| Leu198 | 8.15 | 4.20 (-0.18) | 1.92 | | 1.50 | | 0.90 (Me) | | | |

| | | | | | | | | | |
|---|---|---|---|---|---|---|---|---|---|
| Ser199 | 8.32 | 4.48 (0.02) | | 3.98 | | | | | |
| Gly200 | 8.31 | 3.91 (-0.12) | | | | | | | |
| Met201 | 8.55 | 4.20 (-0.30) | | 2.22 | | | 2.57 | | |
| Glu202 | 8.18 | 4.32 (0.02) | | 2.27 | | | 2.71 | | |
| Gly203 | | | | | | | | | |
| Arg204 | 7.88 | 4.18 (-0.14) | | 1.89 | | | 1.71 | | |
| Lys205 | | | | | | | | | |
| Lys206 | | | | | | | | | |
| Lys207 | | | | | | | | | |
| Phe208 | 8.09 | 4.62 (-0.04) | 3.08 | | 3.30 | | | | 7.30 |
| Glu209 | 8.02 | 4.47 (0.17) | | 2.09 | | 2.24 | | 2.57 | |
| Ser210 | 8.14 | 4.46 (0.08) | | 3.92 | | | | | |

[a] For the $H_\alpha$ proton column, the value within parenthesis is the conformational shift ($\delta_{res}$-$\delta_{rc}$). The random-coil values for the sequence were obtained from: https://spin.niddk.nih.gov/bax/nmrserver/Poulsen_rc_CS/).

Table ST5: Chemical shifts (δ, ppm from TSP) of HcTnI-C27 in aqueous solution in the presence of 45.7 μM of αTm (binding buffer) at 10 ºC (14.1 T)[a]

| | NH | Hα | Hβ2 | Hβ3 | Hγ2 | Hγ3 | Hδ2 | Hδ3 | Hε | Hζ |
|---|---|---|---|---|---|---|---|---|---|---|
| Glu184 | | 3.98 (-0.29) | 2.03 | | 2.30 | | | | | |
| Asn185 | 9.04 | 4.73 (0.08) | 2.72 | | | | | | | |
| Arg186 | 8.66 | 4.23 (-0.10) | 1.72 | | 1.65; 1.55 | | 3.06 | | 6.80; 6.55 | |
| Glu187 | 8.50 | 4.24 (-0.08) | 22.25; 1.84 | | | | | | | |
| Val188 | 8.23 | 4.18 (0.06) | 2.01 | | 0.89 (Me) | | | | | |
| Gly189 | 8.49 | 3.82 (-0.10) | | | | | | | | |
| Asp190 | 8.32 | 4.56 (0.06) | 2.64; 2.70 | | | | | | | |
| Trp191 | 8.01 | 4.48 (-0.19) | 3.32 | | | | 10.19 (NH); 7.25 (2H); 7.44 (7H); 7.16 (6H); 7.08 (5H); 7.48 (4H) | | | |
| Arg192 | 7.76 | 3.84 (-0.31) | 1.59; 1.41 | | 0.90; 0.81 | | 2.89 | | 6.80: 6.55 | |
| Lys193 | 7.90 | 4.06 (-0.08) | 1.72 | | 1.55; 1.35 | | | | | |
| Asn194 | 8.25 | 4.61 (-0.07) | 2.74 | | | | | | | |
| Ile195 | 8.05 | 4.05 (-0.08) | 1.80 | | 1.12 | 1.34 | 0.87 (Me) | | | |
| Asp196 | 8.52 | 4.59 (0.02) | 2.75 | 2.62 | | | | | | |
| Ala197 | 8.25 | 4.23 (-0.08) | 1.36 (Me) | | | | | | | |
| Leu198 | 8.18 | 4.32 (-0.06) | 1.66; 1.35 | | 1.35 | | 0.87 (Me) | | | |
| Ser199 | 8.05 | 4.29 (-0.17) | 3.82 | | | | | | | |

| | | | | | | | | | |
|---|---|---|---|---|---|---|---|---|---|
| Gly200 | 8.45 | 3.94 (-0.09) | | | | | | | |
| Met201 | 8.21 | 4.43 (-0.07) | | 2.02 | | 2.44 | | 2.52 | |
| Glu202 | 8.54 | 4.24 (-0.06) | 1.95 | | 2.00 | 2.36 | | | |
| Gly203 | 8.45 | 3.95 (-0.02) | | | | | | | |
| Arg204 | 8.05 | 4.25 (-0.07) | | 1.80 | | | 1.59 | | 3.14 |
| Lys205 (*) | 8.34 | 4.15 (-0.14) | | 1.58 | | | 1.32 | | |
| Lys206 (*) | 8.31 | 4.20 (-0.02) | | 1.58 | | | 1.32 | | |
| Lys207 (*) | 8.34 | 4.24 (0.00) | | 1.72 | | | 1.39 | | |
| Phe208 | 8.34 | 4.57 (-0.09) | | 3.04 | | | | | 7.19 |
| Glu209 | 8.27 | 4.39 (0.09) | 1.83 | | 2.04 | | 2.25 | | |
| Ser210 | 8.19 | 4.30 (-0.08) | | 3.88 | | | | | |

[a] The (*) indicates those residues whose resonances could not be unambiguously assigned. For the $H_\alpha$ proton column, the value within parenthesis is the conformational shift ($\delta_{res}$-$\delta_{rc}$). The random-coil values for the sequence were obtained from: https://spin.niddk.nih.gov/bax/nmrserver/Poulsen_rc_CS/).

Table ST6: Chemical shifts (δ, ppm from TSP) of mutant HcTnI-C27-H in aqueous solution (pH 4.5 (50 mM sodium acetate buffer), 10 ºC)[a]

| | NH | $H_{\alpha}$ | $H_{\beta 2}$ | $H_{\beta 3}$ | $H_{\gamma 2}$ | $H_{\gamma 3}$ | $H_{\delta 2}$ | $H_{\delta 3}$ | $H_{\varepsilon}$ | $H_{\zeta}$ |
|---|---|---|---|---|---|---|---|---|---|---|
| Glu184 | | 4.04 | 2.08 | | 2.35 | | | | | |
| Asn185 | 9.04 | 4.74 | 2.78 | | | | | | | |
| Arg186 | 8.65 | 4.28 | 1.79 | | 1.56 | | 3.13 | | 7.19 (NH) | |
| Glu187 | 8.57 | 4.20 | 2.04 | | 2.35 | | | | | |
| Val188 | 8.25 | 4.10 | 2.05 | | 0.92 (Me) | | | | | |
| Gly189 | 8.48 | 3.86 | | | | | | | | |
| Asp190 | 8.29 | 4.62 | 2.66 | | | | | | | |
| Trp191 | 8.01 | 4.53 | 3.24 | | | | 10.14 (NH); 7.25 (2H); 7.46 (7H); 7.07 (6H); 7.19 (5H); 7.43 (4H) | | | |
| His192 | 8.12 | 4.28 | 2.85 | 3.06 | | | 6.99 (C4H) | | 8.44 (C2H) | |
| Lys193 | 8.06 | 4.13 | 1.73 | | 1.36 | | | | | |
| Asn194 | 8.44 | 4.60 | 2.76 | | | | | | | |
| Ile195 | 8.16 | 4.11 | 1.88; 0.87 (Me) | | 1.40 | 1.15 | 0.87 (Me) | | | |
| Asp196 | 8.44 | 4.62 | 2.76 | | | | | | | |
| Ala197 | 8.29 | 4.27 | 1.40 (Me) | | | | | | | |
| Leu198 | 8.19 | 4.34 | 1.68 | | | | 0.89 (Me) | | | |
| Ser199 | 8.01 | 4.23 | 3.86 | | | | | | | |

| | | | | | |
|---|---|---|---|---|---|
| Gly200 | 8.45 | 3.93 | | | |
| Met201 | 8.22 | 4.43 | 2.05 | 2.51 | |
| Glu202 | 8.51 | 4.28 | 1.93 | 2.28 | |
| Gly203 | 8.46 | 3.97 | | | |
| Arg204 | 8.04 | 4.28 | 1.77 | 1.65 | 3.17 |
| Lys205 (*) | 8.33 | 4.20 | 1.71 | 1.41 | |
| Lys206 (*) | 8.35 | 4.23 | 1.71 | 1.41 | |
| Lys207 (*) | 8.29 | 4.22 | 1.71 | 1.41 | |
| Phe208 | 8.35 | 4.60 | 3.08 | | 7.23 |
| Glu209 | 8.29 | 4.36 | 2.05 | 2.28 | |
| Ser210 | 8.21 | 4.37 | 3.86 | | |

[a] The (*) indicates those residues whose resonances could not be unambiguously assigned.

Table ST7: Chemical shifts (δ, ppm from TSP) of wild-type HcTnI-C27 in aqueous solution (pH 4.5 (50 mM sodium acetate buffer), 10 ºC)[a]

| | NH | Hα | Hβ2 | Hβ3 | Hγ2 | Hγ3 | Hδ2 | Hδ3 | Hε | Hζ |
|---|---|---|---|---|---|---|---|---|---|---|
| Glu184 | | 4.05 | 2.10 | | 2.35 | | | | | |
| Asn185 | 9.05 | 4.76 | 2.77 | | | | | | | |
| Arg186 | 8.66 | 4.28 | 1.77 | | 1.56 | | 3.12 | | | |
| Glu187 | 8.53 | 4.30 | 1.97 | | 2.25 | | | | | |
| Val188 | 8.24 | 4.11 | 2.09 | | 0.95 (Me) | | | | | |
| Gly189 | 8.50 | 3.89 | | | | | | | | |
| Asp190 | 8.32 | 4.59 | 2.66 | | | | | | | |
| Trp191 | 8.01 | 4.53 | 3.33 | | | | | | 10.21 (NH); 7.33 (2H); 7.48 (7H); 7.17 (6H); 7.15 (5H); 7.53 (4H) | |
| Arg192 | 7.78 | 3.86 | 1.58; 1.41 | | 0.95; 0.88 | | 2.94 | | | |
| Lys193 | 7.90 | 4.12 | 1.75 | | 1.37 | | | | | |
| Asn194 | 8.24 | 4.65 | 2.81 | | | | | | | |
| Ile195 | 8.05 | 4.11 | 1.88; 0.90 (Me) | | 1.18 | 1.39 | 0.90 (Me) | | | |
| Asp196 | 8.41 | 4.56 | 2.68 | | | | | | | |
| Ala197 | 8.24 | 4.23 | 1.39 (Me) | | | | | | | |
| Leu198 | 8.18 | 4.34 | 1.69 | | | | 0.89 (Me) | | | |
| Ser199 | 8.00 | 4.23 | 3.83 | | | | | | | |

| | | | | | |
|---|---|---|---|---|---|
| Gly200 | 8.46 | 3.93 | | | |
| Met201 | 8.21 | 4.41 | 2.02 | 2.55 | |
| Glu202 | 8.58 | 4.20 | 2.03 | 2.34 | |
| Gly203 | 8.44 | 3.97 | | | |
| Arg204 | 8.05 | 4.30 | 1.79 | 1.58 | 3.19 |
| Lys205 (*) | 8.35 | 4.21 | 1.68 | 1.30 | |
| Lys206 (*) | 8.35 | 4.20 | 1.68 | 1.30 | |
| Lys207 (*) | 8.29 | 4.27 | 1.72 | 1.40 | |
| Phe208 | 8.29 | 4.62 | 3.11 | | 7.25 |
| Glu209 | 8.31 | 4.38 | 1.90 | 2.26 | |
| Ser210 | 8.21 | 4.37 | 3.90 | | |

[a] The (*) indicates those residues whose resonances could not be unambiguously assigned.

## SUPPORTING FIGURES

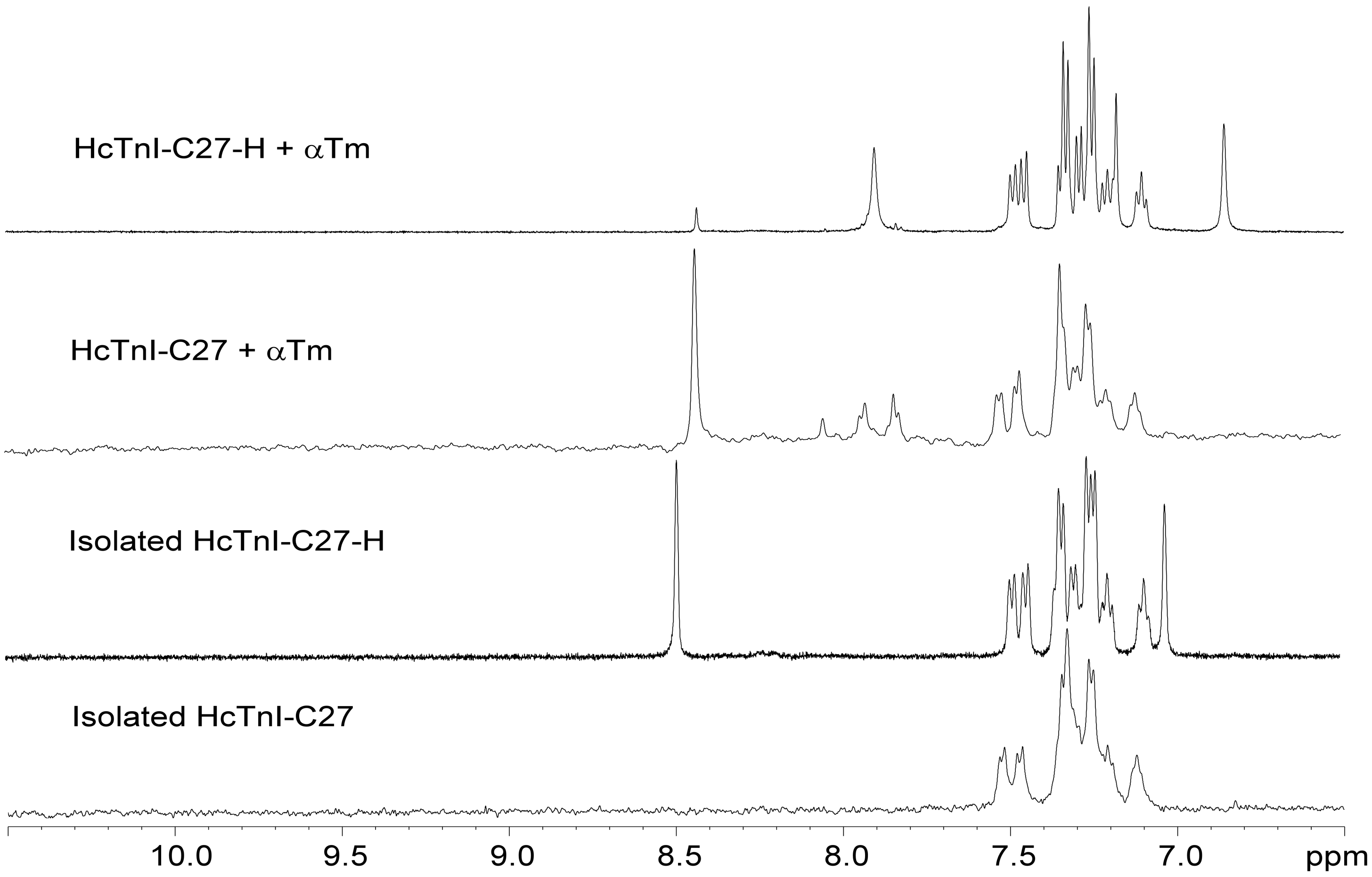


**FIGURE S1: 1D $^{1}$H-NMR spectra of the peptides under different conditions in $D_2O$**: The amide regions of the NMR spectra of the peptides in the absence or in the presence of αTm dissolved in $D_2O$ are shown. Experiments were acquired in binding buffer or in 50 mM sodium phosphate buffer (pH 7.2) at 10 ºC.

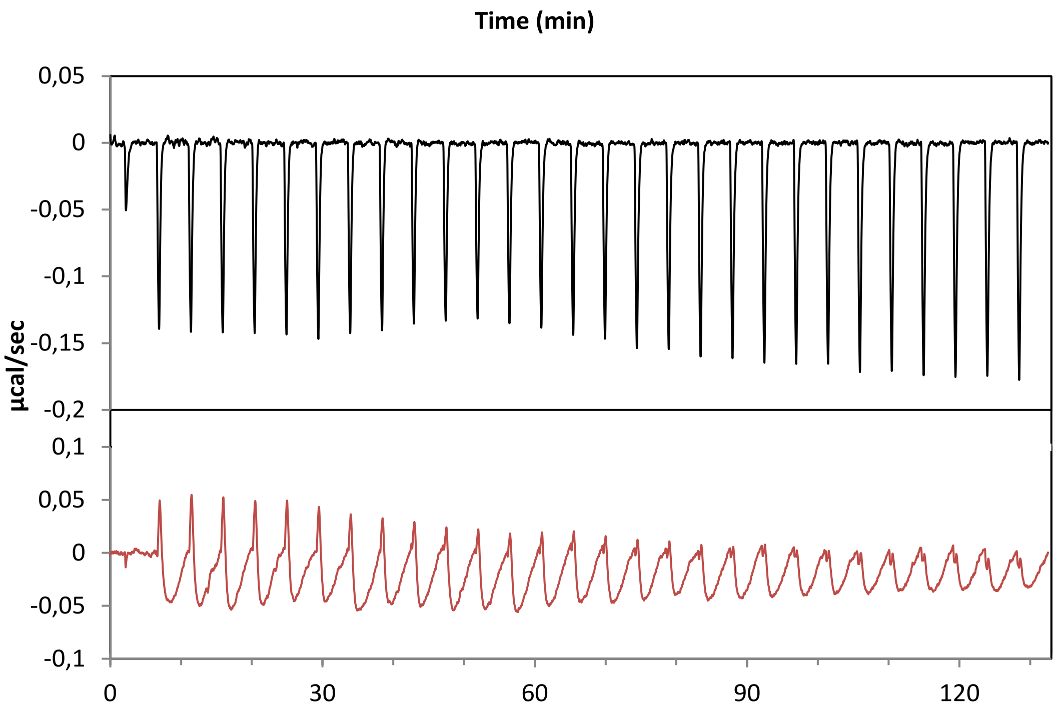


**FIGURE S2: Raw data of the ITC titration of wild-type peptide *versus* αTm**: The titration of the peptide on αTm (black); and the dilution of the isolated peptide (red).

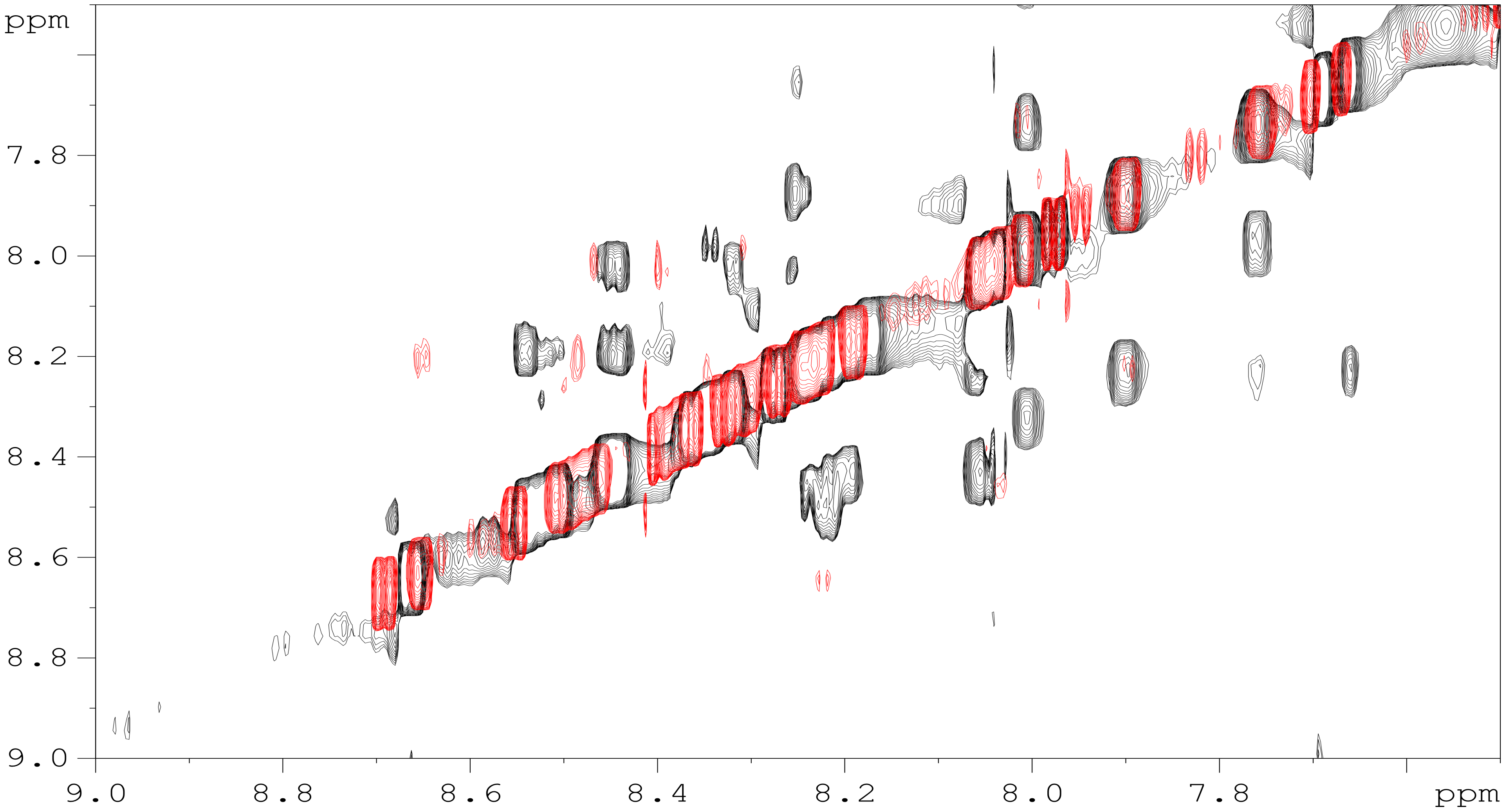


**FIGURE S3: NOESY spectra of HcTnI-C27 in isolation or in the presence of αTm**: (Red) The amide-amide region of the NOESY spectra of isolated HcTnI-C27, and in the presence of micromolar amounts of αTm (black). Experiments were acquired in binding buffer (black) or in 50 mM sodium phosphate buffer (red) and 10 ºC. Both experiments were drawn with the same lowest contour level.

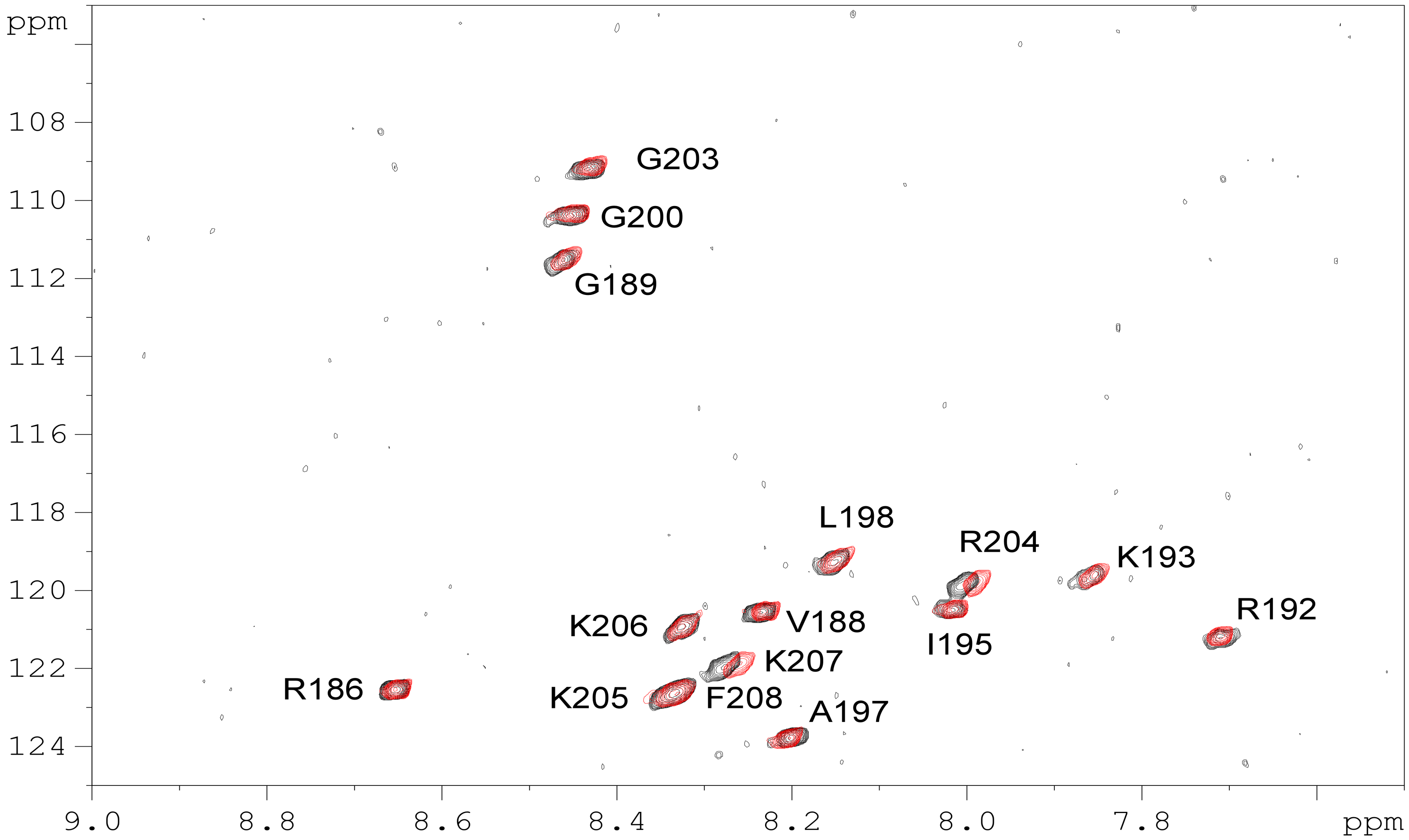


**FIGURE S4: HSQC spectra**: (Red) The HSQC spectrum of isolated HcTnI-C27 (40 µM). (Black) The HSQC spectrum of HcTnI-C27 (20 µM) in the presence of αTm (104 µM). Experiments were acquired in binding buffer (black) or in 50 mM sodium phosphate buffer (red) and 10 ºC. The signals of Ile195 and Arg204 could be resolved in the spectrum of the bound peptide (black), but they are closer than in the spectrum of the free peptide (red); the cross-peak of Phe208 overlaps with that of Lys205. Both experiments were drawn with the same lowest contour level.

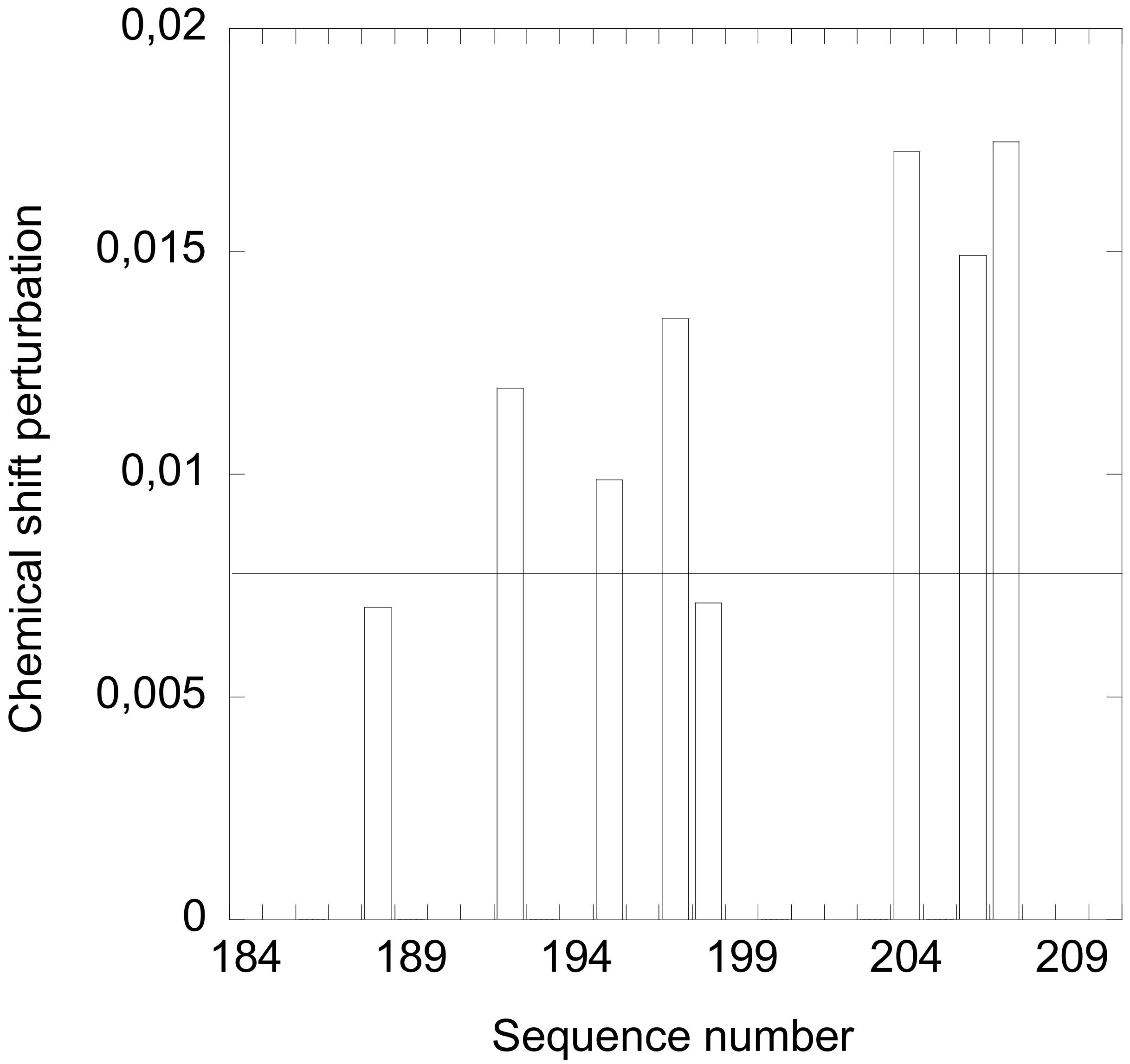


**FIGURE S5: Changes in chemical shifts of HcTnI-C27 upon addition of αTm**: The values of chemical shift perturbation (CSP) were calculated from $CSP = \sqrt{(\Delta\delta_H)^2 + (0.14\Delta\delta_N)^2}$ (see main text for details) obtained from the chemical shifts measured in the HSQC spectra of Fig. S4. The line is the average of the CSPs

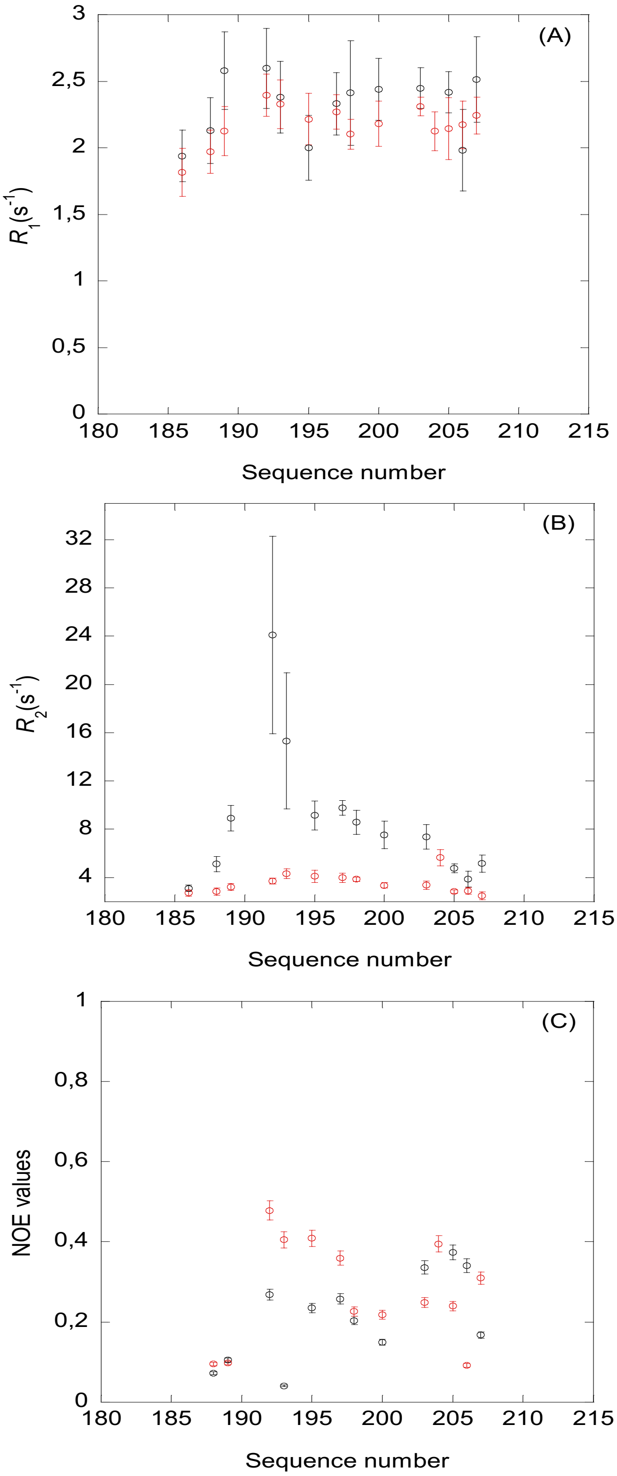


**FIGURE S6: Relaxation parameters of HcTnI-C27**: The $R_1$ values (A), the $R_2$ values (B) and the NOE values of isolated HcTnI-C27 (40 µM) (in red) or in the presence of αTm (104 µM) (in black) at 14.1 T. Experiments were acquired at pH 7.2 in binding buffer (black) or in 50 mM sodium phosphate buffer (red) and 10 ºC. The NOE values of Ile195 under both conditions are probably the sum of those of this residue and Arg204 (as the cross-peaks overlap), and the values of the relaxation rates and NOEs of Lys205 do probably include those of Phe208. For the sake of consistency, we do not include the $R_1$ and the $R_2$ values of Arg204 in the presence of αTm (black points), although we were able to measure them (see main text for details).
